# Dentate gyrus activin signaling mediates the antidepressant response

**DOI:** 10.1038/s41398-020-01156-y

**Published:** 2021-01-07

**Authors:** Mark M. Gergues, Christine N. Yohn, Anusha Bharadia, Marjorie R. Levinstein, Benjamin Adam Samuels

**Affiliations:** 1 Department of Psychology, Rutgers, The State University of New Jersey, New Brunswick, NJ USA; 2 Department of Psychiatry, Columbia University and Research Foundation for Mental Hygiene, New York State Psychiatric Institute, New York, NY USA; 3 Present Address: Neuroscience Graduate Program, Weill Institute for Neurosciences, University of California, San Francisco, San Francisco, CA USA; 4 Present Address: Department of Psychiatry & Behavioral Sciences, University of Washington, Seattle, WA USA

**Keywords:** Neuroscience, Psychiatric disorders

## Abstract

Antidepressants that target monoaminergic systems, such as selective serotonin reuptake inhibitors (SSRIs), are widely used to treat neuropsychiatric disorders including major depressive disorder, several anxiety disorders, and obsessive-compulsive disorder. However, these treatments are not ideal because only a subset of patients achieve remission. The reasons why some individuals remit to antidepressant treatments while others do not are unknown. Here, we developed a paradigm to assess antidepressant treatment resistance in mice. Exposure of male C57BL/6J mice to either chronic corticosterone administration or chronic social defeat stress induces maladaptive affective behaviors. Subsequent chronic treatment with the SSRI fluoxetine reverses these maladaptive affective behavioral changes in some, but not all, of the mice, permitting stratification into persistent responders and non-responders to fluoxetine. We found several differences in expression of Activin signaling-related genes between responders and non-responders in the dentate gyrus (DG), a region that is critical for the beneficial behavioral effects of fluoxetine. Enhancement of Activin signaling in the DG converted behavioral non-responders into responders to fluoxetine treatment more effectively than commonly used second-line antidepressant treatments, while inhibition of Activin signaling in the DG converted responders into non-responders. Taken together, these results demonstrate that the behavioral response to fluoxetine can be bidirectionally modified via targeted manipulations of the DG and suggest that molecular- and neural circuit-based modulations of DG may provide a new therapeutic avenue for more effective antidepressant treatments.

## Introduction

Approximately 32–35 million adults in the US population (16%) experience an episode of major depression in their lifetime^1^, and commonly used treatments, such as selective serotonin reuptake inhibitors (SSRIs), are not ideal since only a subset of patients (~33%) achieves remission with initial treatment^2,3^. However, despite this large population of non-remitters, the reasons why some individuals remit to antidepressant treatments while others do not remain unknown. Given that SSRIs are widely used to treat not only major depressive disorder, but also several anxiety disorders and obsessive-compulsive disorder, improving our understanding of this treatment resistance is of paramount importance. One approach is to decipher the neural circuitry and molecular mechanisms that underlie antidepressant treatment response and resistance.

Several brain regions, including prefrontal and cingulate cortices, amygdala, thalamus, hypothalamus, nucleus accumbens, and hippocampus are implicated in mood disorders through imaging and postmortem studies^4,5^. Within the hippocampus, mounting evidence indicates the dentate gyrus (DG) subfield plays an important role. Humans suffering from the major depressive disorder have fewer DG granule cells (DG GCs) than controls and DG volume is inversely correlated with the number of depressive episodes^6,7^. Several preclinical studies in rodents also demonstrate that DG is an essential component of the neural circuitry mediating the antidepressant response. Serotonin 1A receptors on mature DG granule cells are critical mediators of the behavioral and the neuroendocrine response to the SSRI fluoxetine^8^. Furthermore, chronic treatment with most antidepressants (including SSRIs) stimulates adult neurogenesis in the DG^9,10^. Chronic SSRI treatment increases proliferation of dividing neural precursor cells and promotes maturation and integration of young adult born granule cells (abGCs) into the DG and ablation or impairment of this neurogenic niche results in the loss of some antidepressant-mediated behaviors^9–14^. Targeting entorhinal cortex projections to the DG yields an antidepressant-like behavioral response^15^. Optogenetic and chemogenetic manipulations of ventral DG granule cells demonstrate a role in avoidance behaviors and stress resilience^16–19^. Serotonin 1B receptors on cholecystokinin (CCK) inhibitory interneurons in the DG are also essential for mediating the behavioral response to SSRI treatment^20^. Finally, direct peptide infusions of brain-derived neurotrophic factor (BDNF), vascular endothelial growth factor (VEGF), or Activin A into DG yield an antidepressant-like behavioral response^21–25^.

Activin A is a TGFβ superfamily member, and chronic SSRI treatment induces Activin expression and signaling in DG^21,22^. TGFβ superfamily members are multifunctional cell–cell signaling proteins that play important roles in tissue homeostasis and development and mediate pleiotropic effects from the membrane to nucleus through distinct combinations of type I and II serine/threonine kinase receptors^26^. Activin protein complexes bind to one of two type II receptors, Acvr2a or Acvr2b, which then recruit one of three type I receptors, Acvr1, Acvr1b, or Acvr1c. Activin receptor complexes then activate downstream effectors, known as Smad proteins. Receptor-regulated Smad proteins (mainly Smad2 and Smad3), are phosphorylated by activated type I receptors and form heteromeric complexes with a common partner, Smad4, which translocates into the nucleus to control gene transcription^26^. In addition to direct Activin A DG infusions having antidepressant-like effects on behavior, overexpression of Activin A protein complexes in the forebrain reduces avoidance behavior, while overexpression of an antagonist of Activin signaling yields an increase in avoidance and decreases in adult neurogenesis in DG^27^.

Exposure of rodents to chronic stressful experiences can induce a long-lasting alteration in the affective state in which there are increases in maladaptive affective behaviors. Several highly distinct stress paradigms are commonly used for this purpose, including chronic mild stress, chronic social defeat stress, and chronic administration of glucocorticoids^13,28–40^. Importantly, these stressed rodents can be treated with antidepressants to reverse the maladaptive affective behaviors and better understand the neural effects of antidepressants. Interestingly, we have noticed that in the Novelty Suppressed Feeding (NSF) behavioral task, SSRI treatment only reverses the effects of chronic glucocorticoid administration in a subset of mice, suggesting that there may be responders and non-responders to antidepressant treatment^32,41^. Therefore, we sought to better understand and characterize this treatment resistance phenotype and then to assess differences in the DG between responders and non-responders in order to determine how to manipulate the DG to modify the response to antidepressant treatment.

## Materials and methods

### Mice

Adult 8-week-old male mice purchased from Jackson labs (C57BL/6J) were housed in groups of three to five per cage with *ad libitum* access to food and water. Mice were on a 12:12-h light/dark schedule. All behavioral testing was conducted during the light period and using the same testing time throughout the experiments. For drug administration and social defeat information please see Supplemental Materials. The number of mice needed for each group was based on both a power analyses and previous publications of the CORT+FLX paradigm. We approximated that ~33% of CORT+FLX mice would be non-responders. All experiments were approved by the Institutional Animal Care and Use Committee at either Rutgers or Columbia/NYSPI.

### Stress paradigms

#### Chronic cortiscosterone

Adult male C57BL/6J mice were randomly divided into Corticosterone (CORT) and vehicle (VEH) treatments, with weights measured on a weekly basis during treatment. VEH-treated mice were administered 0.45% beta-cyclodextrin dissolved in their drinking water, whereas CORT-treated mice received a CORT (35 μg/mL) (Sigma-Aldrich, St. Louis, MO) dissolved in 0.45% beta-cyclodextrin (4.5 mg/mL) (Sigma-Aldrich, St. Louis, MO). CORT was administered in opaque bottles due to the light sensitivity of the drug^13^. CORT treatment lasted the duration of all experiments.

#### Social defeat stress

In brief, defeat stress was carried out using similar methods to those already published^17,34,42^. After the 10-day defeat stress protocol, we used the social interaction test to differentiate between susceptible (SUS) and resilient (RES) mice to the social defeat stress. Additional details are provided in the Supplemental Materials.

#### Behavioral testing

Behavioral testing was conducted after 3 weeks of antidepressant administration, in the following order: elevated plus maze (EPM), novelty suppressed feeding (NSF) and then forced swim test (FST). Mice were given 3 days between behavioral tests to avoid contaminating stressors as well as before sacrifice. Prior to each behavioral test mice were acclimated to the room for 30 mins of habituation. For full description of EPM and FST see Supplemental Materials.

*Novelty suppressed feeding*. NSF was performed as described^8,43^. The testing apparatus consisted of a plastic box (50 × 50 × 20 cm), the floor of which was covered with approximately 2 cm of bedding. 18 h before behavioral testing, mice were weighed and then food deprived. At the time of testing, a single pellet of food was placed on a white paper platform in the center of the box beneath a gooseneck lamp illuminating the center of the area at about 1500 lux. A mouse was placed in a corner of the box and latency to approach and eat the food pellet was recorded with a maximum of 10 min. Immediately after the testing period, the mice were transferred to their home cages and latency to feed in the home cage as well as consumption was recorded. After completing testing, animals were weighed again and % change in body weight was calculated with respect to pre-testing weights. We controlled for all NSF behavior by also assessing home cage consumption.

#### Gene expression

Animals were sacrificed via rapid decapitation and ventral DG was microdissected, flash frozen, and then stored at −80 °C until further processing. RNA was extracted from tissue samples using a RNA/DNA Purification kit (Norgen Biotek). Total RNA was then converted into cDNA using Superscript III enzyme (Invitrogen). Quantitative-PCR was performed in triplicate reactions with Taqman Fast Advanced Mastermix and Taqman probes for activin a, acvr1a, acvr1b, acvr1c, smad2, smad3, and rn18s (Life Technologies) on a StepOne Plus Real-Time PCR System (Applied Biosystems). Data were analyzed using the ΔΔC_T_ method, triplicate cycle thresholds per gene per sample were averaged, normalized to control gene (*rn18s*) to obtain ΔC_T_, and were then converted to ΔΔC_T_ values by normalizing to mean ΔC_T_’s of the vehicle group. Final values were then expressed as an expression percentage relative to the vehicle group values.

#### Intracerebral infusions

Mice were anesthetized with sodium pentobarbital (diluted 1:10 from stock of 50 mg/ml and injected at a volume of 10 ml/kg) (Henry Schein) and guide cannulae with dummy cannulae (Plastics 1) were implanted. For ventral DG the coordinates used were: −3.5 mm and ±2.8 mm from bregma at a depth of 3.6 mm from the skull surface, and for CA1 the coordinates used were: −3.1 mm and ±3.0 mm from the bregma at a depth of 2.0 mm from the skull surface. 1–2 weeks after surgery, animals began to receive bilateral infusions of either vehicle (0.1% BSA in sterile PBS, pH 7.4) (Sigma-Aldrich), 1μg of mouse Activin A peptide (R&D Systems), 1 μg of mouse Inhibin A peptide (R&D Systems), or 1μg each of Activin A + Inhibin A (R&D Systems) in sterile PBS, pH 7.4 once per day (over a time course of 15 min per side, 10 min of infusion and an additional 5 min with tubing left in place) for 2 weeks prior to behavioral testing. Each day connector assemblies with tubing were connected to internal cannulae, which were then inserted into the guide cannulae. Infusions were delivered by a standard infusion only syringe pump (Harvard Apparatus). A total volume of 1.0 μl was infused in each hemisphere per day. Animals were freely moving in their cage during infusions.

#### Statistics

All statistics were performed using Prism software (Graphpad). NSF data were used to determine the bimodal distribution of behavior phenotypes. The D’Agostino & Pearson Omnibus Normality Test was used to establish that these data were not normally distributed and were bimodal as previously described^44^. Parametric hypotheses were assessed with parametric tests. Two-way ANOVAs assessing stress pretreatment x antidepressant treatment were used for EPM, FST, Negative Affect Index, and gene expression. One-way ANOVAs were then subsequently used to assess Stress+VEH, Stress+FLX responders, and Stress+FLX non-responders. NSF was assessed using non-parametric Kaplan–Meier survival analysis with log-rank Mantel–Cox. Correlational analysis between individual animal behavioral values (NSF and either EPM or FST) was performed using Pearson *r* and best-fit values with a linear regression analysis and slopes with analysis significant non-zeroes were analyzed. Post hoc Bonferroni corrections were used where appropriate.

**Also see** Supplemental Materials and Methods

## Results

### Behavioral responders and non-responders to FLX treatment following CORT administration

**Fig. 1:**
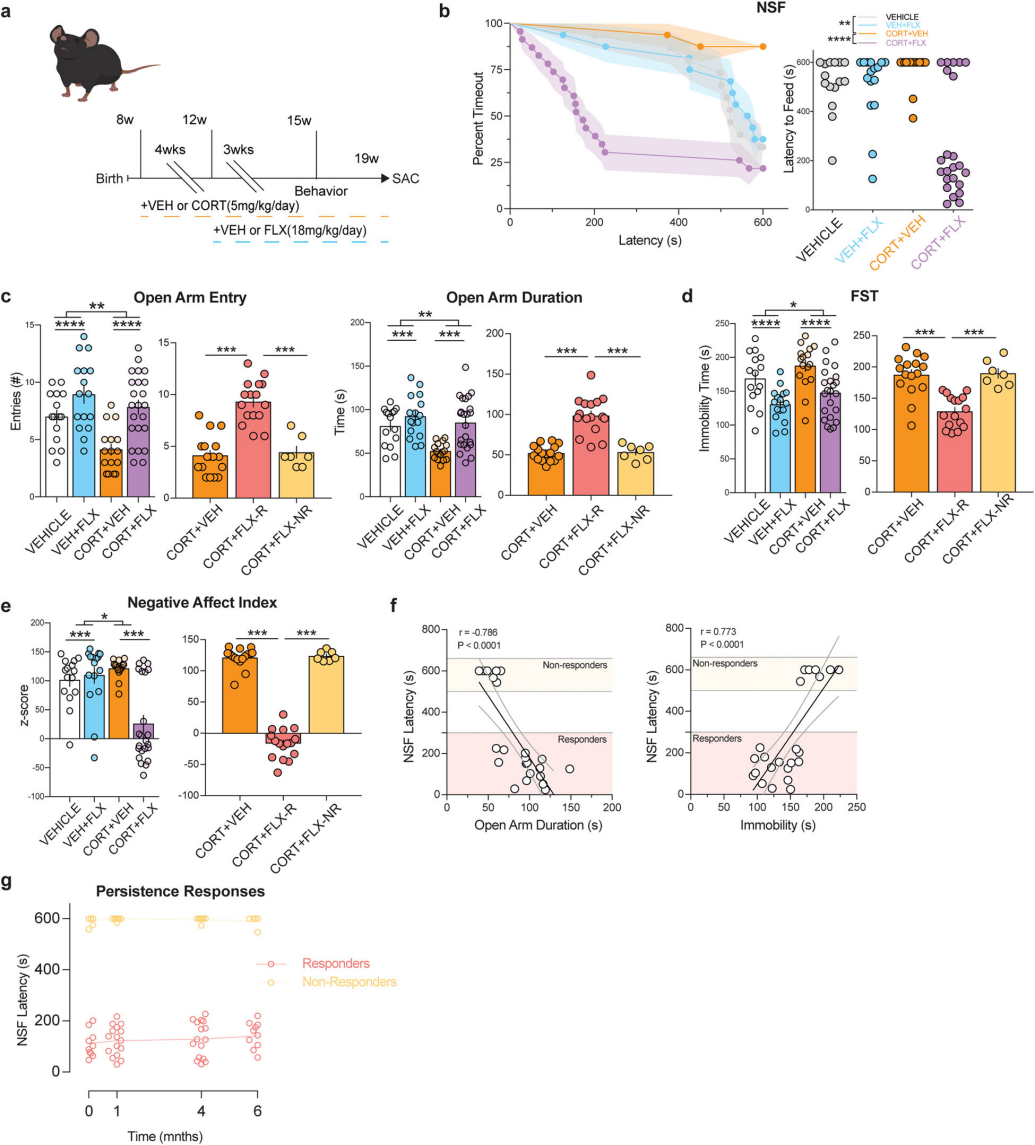
Behavioral responders and non-responders to FLX treatment following CORT administration. **a** Timeline of experiment. **b** Kaplan–Meier survival curve (left) and scatterplot (right) of NSF data showing individual latency to eat values across all four groups. **c**–**e** Left panels represent Two-way ANOVA of all treatment groups and right panels represent One-Way ANOVA of CORT+VEH, CORT+FLX responders, and CORT+FLX non-responders for: EPM open arm entries and open arm duration (**c**), FST immobility (**d**), and Negative Affect Index (**e**). **f** Regression analyses correlating NSF latency to eat with EPM open arm duration (left) and FST immobility (right). **g** In a separate cohort of CORT+FLX mice, persistence of response was determined by assessing NSF behavior after 3 weeks of FLX (time point 0), and then again 1, 4, and 6 months later. For the NSF survival curve, line shading shows SEM of each group (*n* = 15–23 per group). Scatterplots, horizontal lines, and bars show group means with error bars indicating SEM (*n* = 7–16 per group after CORT+FLX mice are divided into responders and non-responders).

To better understand the treatment resistance phenotype following chronic stress and antidepressant treatment, we began by exposing a cohort (*n* = 70) of group housed 8-week-old male C57BL/6J mice to chronic administration with either vehicle or corticosterone (CORT, 5 mg/kg/day via drinking water). Chronic CORT administration at this dosage induces several maladaptive avoidance behaviors, including increased latency to feed in NSF and decreased open arm entries and duration in the elevated plus maze (EPM)^13^. We administered vehicle or CORT for 4 weeks and then added either vehicle or the SSRI fluoxetine (FLX, Prozac, 18 mg/kg/day) for an additional 3 weeks (timeline in Fig. 1a). As expected, chronic CORT increased latency to feed in NSF relative to vehicle only treated mice (CORT+VEH vs VEH *p* = 0.004, log-rank Mantel–Cox test with Bonferroni correction) and coadministration of CORT and FLX significantly reduced latency to feed relative to CORT-treated mice indicative of an antidepressant response (CORT+FLX vs CORT+VEH, *p* < 0.0001, log-rank Mantel–Cox with Bonferroni correction) (Fig. 1b left). However, closer inspection of the individual latencies of CORT+FLX mice demonstrated a bimodal distribution (D’Agostino & Pearson normality test, *p* = 0.0285, Fig. 1b right), providing a potential basis for dividing mice into responders and non-responders to FLX treatment groups. Importantly, responders and non-responders to CORT+FLX show similar levels of FLX in their serum (Supplemental Fig. 1). Furthermore, food consumption in the home cage was similar among all mice (Supplemental Fig. 2).

We next exposed the same cohort of C57BL/6J mice to EPM and then the forced swim test (FST), which is a commonly used test of antidepressant efficacy. In the EPM, separate two-way ANOVAs revealed expected effects of CORT administration and FLX treatment in open arm entries (CORT: *F*_(1,66)_ = 9.69, *p* = 0.0027, FLX: *F*_(1,66)_ = 19.4, *p* < 0.0001) and open arm duration (CORT: *F*_(1,66)_ = 10.34, *p* = 0.002, FLX: *F*_(1,66)_ = 15.42, *p* = 0.0002) (Fig. 1c left panels). To investigate behavioral differences between CORT only treated mice, NSF-defined CORT+FLX responders, and non-responders in the EPM, we next used one-way ANOVAs and found significant differences in open arm entries (*F*_(2,36)_ = 33.24, *p* < 0.001) and duration (*F*_(2,36)_ = 36.54, *p* < 0.001) (Fig. 1c right panels), with Bonferroni-corrected post hoc tests demonstrating that responders had significantly increased open arm entries and duration relative to vehicle-treated mice and non-responders (entries and duration: CORT+VEH vs CORT+FLX-R and CORT+FLX-R vs CORT+FLX-NR, all *p* < 0.001). Non-responders did not show any significant differences relative to vehicle-treated mice (entries and duration: CORT+VEH vs CORT+FLX-NR, *p* > 0.999 for both). These data suggest that FLX response status is conserved across the NSF and EPM. Similarly, in the FST, a two-way ANOVA revealed effects of both CORT administration (*F*_(1,66)_ = 4.83, *p* = 0.031) and FLX treatment (*F*_(1,66)_ = 22.24, *p* < 0.0001) (Fig. 1d left panel) on immobility over the last 4 min of the 6-min test. A separate one-way ANOVA demonstrated significant differences in immobility (*F*_(2,36)_ = 21.02, *p* < 0.001) (Fig. 1d right panel), with Bonferroni-corrected post hoc tests demonstrating that responders had significantly decreased immobility relative to vehicle-treated mice and non-responders (CORT+VEH vs CORT+FLX-R and CORT+FLX-R vs CORT+FLX-NR, both *p* < 0.001). Non-responders did not show any significant differences relative to vehicle-treated mice in the FST (CORT+VEH vs CORT+FLX-NR, *p* > 0.999). Taken together, these data suggest that FLX response status across NSF, EPM, and FST is conserved in CORT-treated mice.

A Negative Affect Index was next used to assess the behavior of this cohort of mice across EPM, NSF, and FST as previously described^45,46^ (Fig. 1e). Briefly, z-scores were calculated in each behavioral test (EPM, NSF, FST) by normalizing individual animals against control group averages and standard deviations. Each behavioral test z-score was then averaged for each animal and group averages were calculated. The score shows a more comprehensive analysis of behavior across multiple behavioral modalities where a score above zero represents maladaptive behaviors (low open arm entries and time in the EPM, high immobility times in the FST, and longer latency to feed in the NSF) task relative to control. A two-way ANOVA revealed significant effects of CORT (*F*_(1,66)_ = 6.41, *p* = 0.013) and FLX (*F*_(1,66)_ = 12, *p* = 0.0009) treatment on the Negative Affect Index (Fig. 1e left panel). A separate one-way ANOVA demonstrated significant differences in Negative Affect Index (*F*_(2,36)_ = 261.4, *p* < 0.001), with Bonferroni-corrected post hoc tests demonstrating that responders had a significantly decreased Negative Affect Index relative to vehicle-treated mice and non-responders (CORT+VEH vs CORT+FLX-R and CORT+FLX-R vs CORT+FLX-NR, both *p* < 0.001) (Fig. 1e right panel). Non-responders did not have a significantly different Negative Affect Index than vehicle-treated mice (CORT+VEH vs CORT+FLX-NR, *p* > 0.999).

We next directly assessed the relationship between NSF latency to feed and behavioral performance in the EPM and FST among CORT+FLX-treated mice. Significant relationships emerged between NSF latency to feed and open arm time (Pearson *r* = −0.786, *p* < 0.0001, Fig. 1f), as well as NSF latency to feed and immobility duration (Pearson *r* = 0.773, *p* < 0.0001). To characterize these relationships further we ran two separate linear regressions, with NSF latency and open arm time having a linear regression line (*y* = −6.02*x* + 780, *F*_(1,21)_ = 33.9, *p* < 0.0001), and FST linear regression line (*y* = 4.63*x*−415, *F*_(1,21)_ = 31.1, *p* < 0.0001) (Fig. 1f).

Finally, we wanted to assess whether the responder and non-responder phenotypes persisted (Fig. 1g). To this end, we exposed a new cohort of C57BL/6J mice to CORT+FLX and then assessed behavior in NSF. Similar to the previous cohort, the CORT+FLX mice displayed a bimodal distribution of latencies to feed. These mice then remained on CORT+FLX and were retested several times in the NSF. Importantly, the responder vs non-responder behavioral distinction persists for at least 6 months (repeated measures ANOVA reveals a significant effect of response status [*F*_(1, 22)_ = 4350, *p* < 0.0001], but not time [*F*_(3, 66)_ = 0.255, *p* = 0.8573]). Therefore, the CORT+FLX paradigm permits the definition of persistent responders and non-responders to FLX treatment and potentially allows for additional manipulations in attempts to convert non-responders into responders.

### DG mRNA expression of Activin signaling components correlates with behavioral response to FLX treatment following CORT administration

We previously published a microarray study assessing DG gene expression in CORT+VEH and CORT+FLX-treated mice^41^. However, when we looked at CORT+FLX responders vs non-responders in these microarray data, pathway analyses suggested that there were differences in multiple components of DG Activin signaling. We were particularly interested in further analyzing this pathway because previous reports have demonstrated that some Activin signaling components are altered in DG by antidepressant treatment and that acute Activin A infusions into DG have antidepressant-like effects in FST^21,22^.

**Fig. 2:**
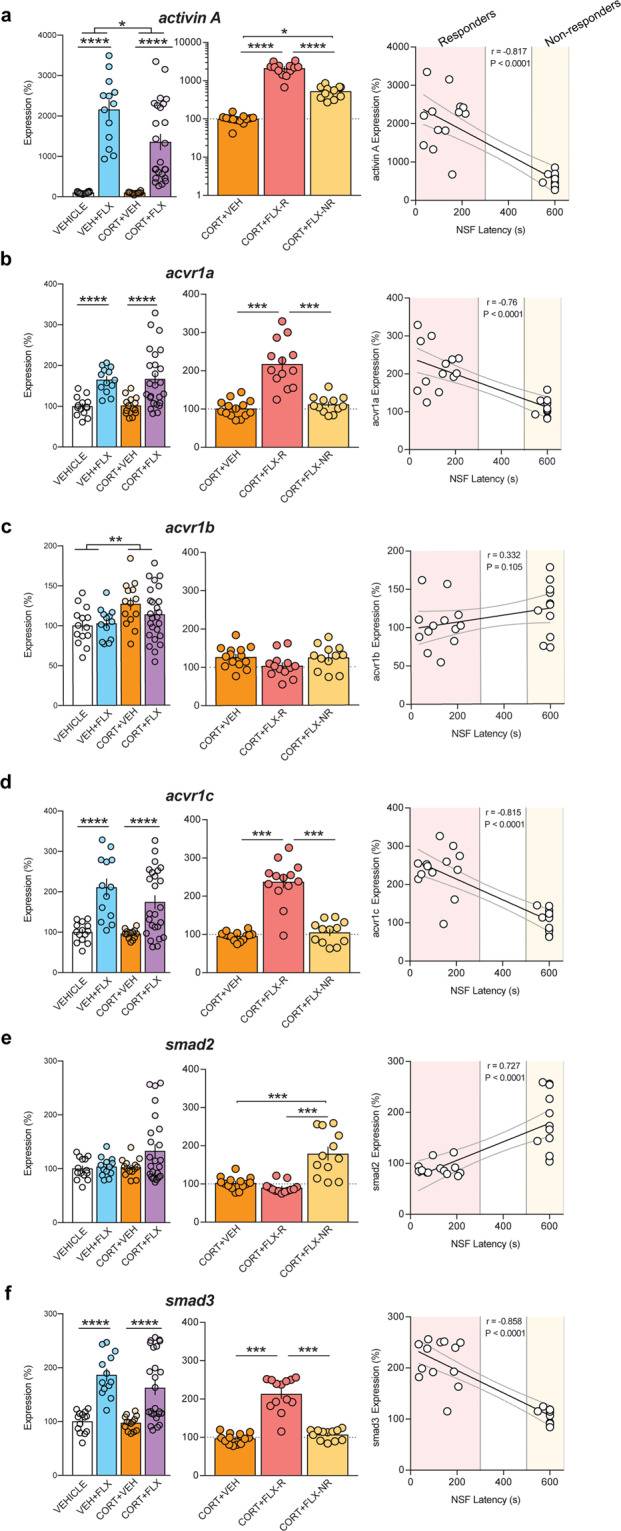
Dentate Gyrus mRNA expression of Activin signaling components correlates with behavioral response to FLX treatment following CORT administration. **a**–**f** Left panels represent Two-way ANOVA of all treatment groups and middle panels represent One-Way ANOVA of CORT+VEH, CORT+FLX responders, and CORT+FLX non-responders for DG mRNA expression of: Activin A (**a**), the Activin receptors acvr1a (**b**), acvr1b (**c**) and acvr1c (**d**), and the intracellular signaling proteins smad2 (**e**) and smad3 (**f**). Right panels show regression analyses correlating NSF latency to eat with DG mRNA expression of: Activin A (**a**), the Activin receptors acvr1a (**b**), acvr1b (**c**) and acvr1c (**d**), and the intracellular signaling proteins smad2 (**e**) and smad3 (**f**). Scatterplots, horizontal lines, and bars show group means with error bars indicating SEM (*n* = 12–14 per group).

To fully characterize Activin signaling in DG of responders and non-responders, we prepared a new cohort of VEH+VEH, VEH+FLX, CORT+VEH, and CORT+FLX-treated mice (behavioral data in Supplemental Fig. 1), and prepared DG RNA following behavioral testing. Two-way ANOVAs revealed significant effects of FLX treatment on DG expression of Activin A (Fig. 2a left panel, *F*_(1,62)_ = 85.51, *p* < 0.0001), the Activin receptors acvr1a (Fig. 2b left panel, *F*_(1,62)_ = 28.15, *p* < 0.0001) and acvr1c (Fig. 2d left panel, *F*_(1,62)_ = 35.37, *p* < 0.0001), and the intracellular signaling protein smad3 (Fig. 2f left panel, *F*_(1,62)_ = 45.19, *p* < 0.0001) and of CORT administration on Activin A (Fig. 2a left panel, *F*_(1,62)_ = 5.01, *p* = 0.0288) and acvr1b (Fig. 2c left panel, *F*_(1,62)_ = 7.285, *p* = 0.009). Separate one-way ANOVAs were next used to compare DG expression of these genes in CORT+FLX responders, CORT+FLX non-responders, and CORT+VEH mice. These analyses revealed significant group differences in Activin A (Fig. 2a middle panel, *F*_(2,36)_ = 81.68, *p* < 0.001), acvr1a (Fig. 2b middle panel, *F*_(2,36)_ = 34.7, *p* < 0.001), acvr1c (Fig. 2d middle panel, *F*_(2,36)_ = 56.97, *p* < 0.001), smad2 (Fig. 2e middle panel, *F*_(2,36)_ = 23.73, *p* < 0.001), and smad3 (Fig. 2f middle panel, *F*_(2,36)_ = 72.33, *p* < 0.001). Interestingly, CORT+FLX responders showed increased expression of Activin A (Fig. 2a), acvr1a (Fig. 2b), acvr1c (Fig. 2d), and smad3 (Fig. 2f) (*p* < 0.001 for all, Bonferroni corrected) relative to CORT only treated mice and non-responders to CORT+FLX. When comparing CORT+FLX non-responders to CORT+VEH mice, we found a significant difference in activin A (*p* = 0.047, Bonferroni corrected) and smad2 expression (*p* < 0.001, Bonferroni corrected), but not in acvr1a, acvr1b, acvr1c, or smad3 expression (all *p* > 0.999, Bonferroni corrected). Finally, we directly compared NSF latency to feed with the DG expression of these genes. Significant relationships emerged between NSF latency to feed and expression of activin A (Pearson *r* = −0.817, *p* < 0.0001), with linear regression line (*y* = −3.23*x* + 2480, *F*_(1,23)_ = 46.2, *p* < 0.0001) (Fig. 2a right panel), acvr1a (Pearson *r* = −0.76, *p* < 0.0001), with linear regression line (*y* = −0.219*x* + 243, *F*_(1,25)_ = 31.5, *p* < 0.0001) (Fig. 2b right panel), acvr1c (Pearson *r* = −0.815, *p* < 0.0001), with linear regression line (*y* = −0.271*x* + 269, *F*_(1,23)_ = 49.9, *p* < 0.0001) (Fig. 2d right panel), smad2 (Pearson *r* = 0.727, *p* < 0.0001), with linear regression line (*y* = 0.108*x* + 69.3, *F*_(1,23)_ = 49.9, *p* < 0.0001) (Fig. 2e right panel), and smad3 (Pearson *r* = −0.858, *p* < 0.0001), with linear regression line (*y* = −0.221*x* + 240, *F*_(1,23)_ = 49.9, *p* < 0.0001) (Fig. 2f right panel). Taken together, all these data demonstrate that DG Activin signaling is significantly different between responders and non-responders to FLX treatment.

### Responders and non-responders to FLX treatment following chronic social defeat stress replicate the behavioral and DG Activin signaling expression data from the CORT administration paradigm

**Fig. 3:**
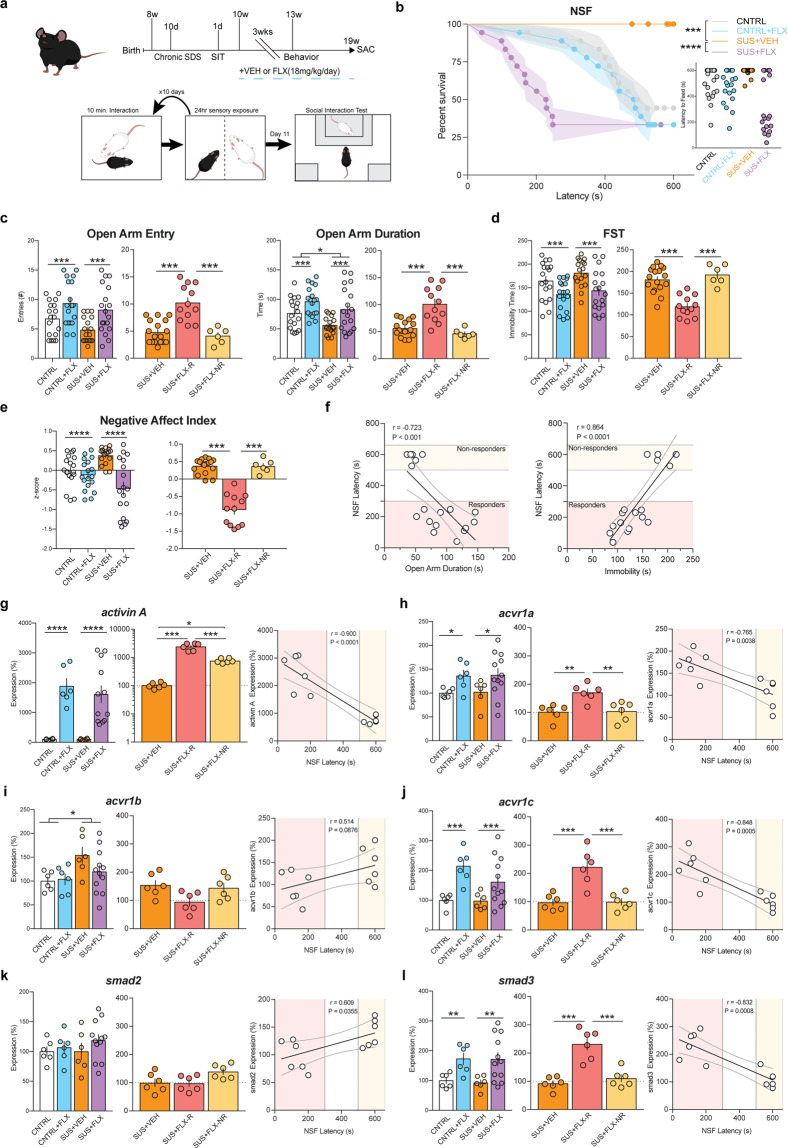
CSDS exposure leads to behavioral responders and non-responders and altered DG Activin signaling. **a** Timeline of experiment and diagram of CSDS and Social Interaction (SIT) paradigms. **b** Kaplan–Meier survival curve (left) and scatterplot (right) of NSF data showing individual latency to eat values across all four treatment groups. **c**–**e** Left panels represent Two-way ANOVA of all treatment groups and right panels represent One-Way ANOVA of CORT+VEH, CORT+FLX responders, and CORT+FLX non-responders for: EPM open arm entries and open arm duration (**c**), FST immobility (**d**), and Negative Affect Index (**e**). **f** Regression analyses correlating NSF latency to eat with EPM open arm duration (left) and FST immobility (right). (g-l) Left panels represent Two-way ANOVA of all treatment groups and middle panels represent One-Way ANOVA of CORT+VEH, CORT+FLX responders, and CORT+FLX non-responders for DG mRNA expression of: Activin A (**g**), the Activin receptors acvr1a (**h**), acvr1b (**i**), and acvr1c (**j**), and the intracellular signaling proteins smad2 (**k**) and smad3 (**l**). Right panels show regression analyses correlating NSF latency to eat with DG mRNA expression of: Activin A (**g**), the Activin receptors acvr1a (**h**), acvr1b (**i**) and acvr1c (**j**), and the intracellular signaling proteins smad2 (**k**) and smad3 (**l**). For survival curves, line shading shows SEM of each group (*n* = 12–14 per group). Scatterplots, horizontal lines, and bars show group means with error bars indicating SEM (*n* = 6–18 per group).

To confirm that these effects on behavior and Activin signaling were due to differential responses to FLX treatment and not a direct or secondary effect of CORT administration, we next repeated these experiments using a distinct chronic stress paradigm. Chronic social defeat stress (CSDS) is a widely used stress paradigm that involves exposing mice to multiple daily defeats by a conspecific from a larger, more aggressive strain^42^. To this end, we exposed a large cohort (*n* = 125) of 8-week-old male C57BL/6J mice to 10 days of either control or CSDS by CD1 male mice prescreened for aggressive behavior (timeline in Fig. 3a). The C57BL/6J mice exposed to CSDS interacted with CD1 aggressors for 5 min per day and then were cohoused with the CD1 aggressors separated by a transparent divider for further sensory exposure. Following the 10 days of control or CSDS, the C57BL/6J mice were next exposed to a social interaction test, which indicated that *n* = 35 of the CSDS exposed mice were susceptible (SUS) to the CSDS (Supplemental Fig. 3). Control and SUS mice were next administered either VEH or FLX (18 mg/kg/day) for 3 weeks and then exposed to NSF, EPM, and FST. Similar to CORT, SUS mice had an increased latency to feed in NSF relative to control (SUS+VEH vs VEH, *p* = 0.0003, log-rank Mantel–Cox test with Bonferroni correction) (Fig. 3b left) and administration of FLX to SUS mice significantly reduced latency to feed (SUS+FLX vs SUS+VEH, *p* < 0.0001, log-rank Mantel–Cox test with Bonferroni correction). Interestingly, similar to CORT, the individual latencies of the SUS+FLX mice showed a bimodal distribution indicative of responders and non-responders to FLX treatment (Fig. 3b right).

The same cohort of mice was next exposed to EPM and FST, and two-way ANOVAs revealed significant effects of FLX treatment for EPM open arm entries (*F*_(1,67)_ = 14.6, *p* = 0.0003) and duration (*F*_(1,67)_ = 13.35, *p* = 0.0005) (Fig. 3c left panels), and FST immobility (*F*_(1,67)_ = 14.68, *p* = 0.00030) (Fig. 3d left panel), and of CSDS on EPM open arm duration (*F*_(1,67)_ = 6.993, *p* = 0.0102) (Fig. 3c left panel). Subsequent one-way ANOVAs to assess responders and non-responders revealed significant differences in EPM open arm entries (*F*_(2,32)_ = 20.17, *p* < 0.001) and open arm duration (*F*_(2,32)_ = 19.12, *p* < 0.001) (Fig. 3c right panels), and FST immobility (*F*_(2,32)_ = 23.34, *p* < 0.001) (Fig. 3d right panel), with SUS+FLX responders showing increased EPM open arm entries and duration and decreased FST immobility relative to SUS+FLX non-responders and SUS+VEH mice (*p* < 0.001 for all, Bonferroni corrected). SUS+FLX non-responders were not significantly different than SUS+VEH mice in EPM open arm entries, EPM open arm duration, and FST immobility (*p* > 0.999 for all, Bonferroni corrected). A two-way ANOVA assessing the Negative Affect Index in this cohort of mice demonstrated a significant effect of FLX treatment (*F*_(1,67)_ = 18.8, *p* < 0.0001) (Fig. 3e left panel). The subsequent one-way ANOVA assessing responders and non-responders found significant group differences (*F*_(2,32)_ = 64.66, *p* < 0.001) (Fig. 3e right panel), with SUS+FLX responders showing a decreased Negative Affect Index relative to SUS+VEH and SUS+FLX non-responders (*p* < 0.001 for both, Bonferroni corrected). SUS+FLX non-responders were not significantly different than SUS+VEH mice (*p* > 0.999, Bonferroni corrected). Significant relationships also emerged when we directly compared NSF latency to feed to EPM open arm duration (Pearson *r* = −0.723, *p* = 0.0007), with linear regression line (*y* = −4.05*x* + 641, *F*_(1,16)_ = 17.5, *p* = 0.0007), and to FST immobility (Pearson *r* = 0.864, *p* < 0.0001), with linear regression line (*y* = 4.22*x*−302, *F*_(1,16)_ = 47.2, *p* < 0.0001) (Fig. 3f). Taken together, these data replicate the CORT behavioral data and demonstrate that FLX response status across NSF, EPM, and FST is conserved in mice susceptible to CSDS.

We next assessed mRNA expression of Activin signaling components in the DG of the CSDS cohort of mice. Two-way ANOVAs revealed significant effects of FLX treatment on DG expression of Activin A (Fig. 3g left panel, *F*_(1,26)_ = 37.45, *p* < 0.0001), acvr1a (Fig. 3h left panel, *F*_(1,26)_ = 7.717, *p* = 0.0127), acvr1c (Fig. 3j left panel, *F*_(1,26)_ = 15.58, *p* = 0.0005), and smad3 (Fig. 3l left panel, *F*_(1,26)_ = 12.64, *p* = 0.0015) and of CSDS on acvr1b (Fig. 3i left panel, *F*_(1,26)_ = 5.808, *p* = 0.023). Subsequent one-way ANOVAs assessing responders and non-responders found significant group differences for Activin A (Fig. 3g middle panel, *F*_(2,15)_ = 54.65, *p* < 0.001), acvr1a (Fig. 3h middle panel, *F*_(2,15)_ = 9.82, *p* = 0.002), acvr1b (Fig. 3i middle panel, *F*_(2,15)_ = 3.78, *p* = 0.047), acvr1c (Fig. 3j middle panel, *F*_(2,15)_ = 16.24, *p* < 0.001), smad2 (Fig. 3k middle panel, *F*_(2,15)_ = 3.769, *p* = 0.047), and smad3 (Fig. 3l middle panel, *F*_(2,15)_ = 23.38, *p* < 0.001). Interestingly, SUS+FLX responders showed increased expression of Activin A (Fig. 3g), acvr1a (Fig. 3h, SUS+VEH vs SUS+FLX-R (*p* = 0.004), SUS+FLX-R VS SUS+FLX-NR (*p* = 0.006)), acvr1c (Fig. 3j), and smad3 (Fig. 3l) (all Bonferroni corrected, *p* < 0.001 for all except acvr1a) relative to SUS+VEH mice and SUS+FLX non-responders. Similar to CORT, the expression of acvr1a, acvr1c, and smad3 were not significantly different between SUS+FLX non-responders and SUS+VEH mice (*p* > 0.999 for all, Bonferroni corrected). Activin A expression was significantly increased in SUS+FLX non-responders relative to SUS+VEH mice (*p* = 0.035, Bonferroni corrected).

Finally, we directly compared NSF latency to feed with DG expression of these genes in responders and non-responders. Significant relationships emerged between NSF latency to feed and expression of activin A (Pearson *r* = −0.900, *p* < 0.0001), with linear regression line (*y* = −3.70*x* + 2920, *F*_(1,10)_ = 42.5, *p* < 0.0001) (Fig. 3g right panel), acvr1a (Pearson *r* = −0.765, *p* = 0.0038), with linear regression line (*y* = −0.145*x* + 189, *F*_(1,10)_ = 14.1, *p* = 0.0038) (Fig. 3h right panel), acvr1c (Pearson *r* = −0.848, *p* = 0.0005), with linear regression line (*y* = −0.278*x* + 259, *F*_(1,10_ = 25.7, *p* = 0.0005) (Fig. 3j right panel), and smad3 (Pearson *r* = −0.832, *p* = 0.0008), with linear regression line (*y* = −0.261*x* + 263, *F*_(1,10)_ = 22.4, *p* = 0.0008) (Fig. 3l right panel). Taken together, these data replicate the CORT Activin data and demonstrate that DG Activin signaling is significantly different between responders and non-responders to FLX treatment. Furthermore, in two distinct stress paradigms, FLX response status is conserved across behavior and DG Activin signaling.

### Chronic Activin A infusions into DG convert FLX non-responders into responders

Since our gene expression data indicate that several components of DG Activin signaling, including Activin A itself, are decreased in FLX non-responders relative to responders, we wanted to test whether this altered signaling underlies the lack of behavioral response to FLX. Acute Activin A infusions directly into DG yield an antidepressant-like response in FST^21,22^, so we reasoned that the development of a chronic Activin A infusion paradigm into DG could potentially convert non-responders to FLX into responders. To this end, we first assessed DG Activin A infusions using a dose response in naïve mice (Supplemental Fig. 4) and then assessed DG Activin A infusions in VEH or CORT-treated mice in the absence of FLX (Supplemental Fig. 5). Like the previously published acute dose, we found that daily bilateral 1.0ug Activin A infusions into DG for two weeks had antidepressant-like effects on behavior and adult neurogenesis (Supplemental Figs. 4–6).

**Fig. 4:**
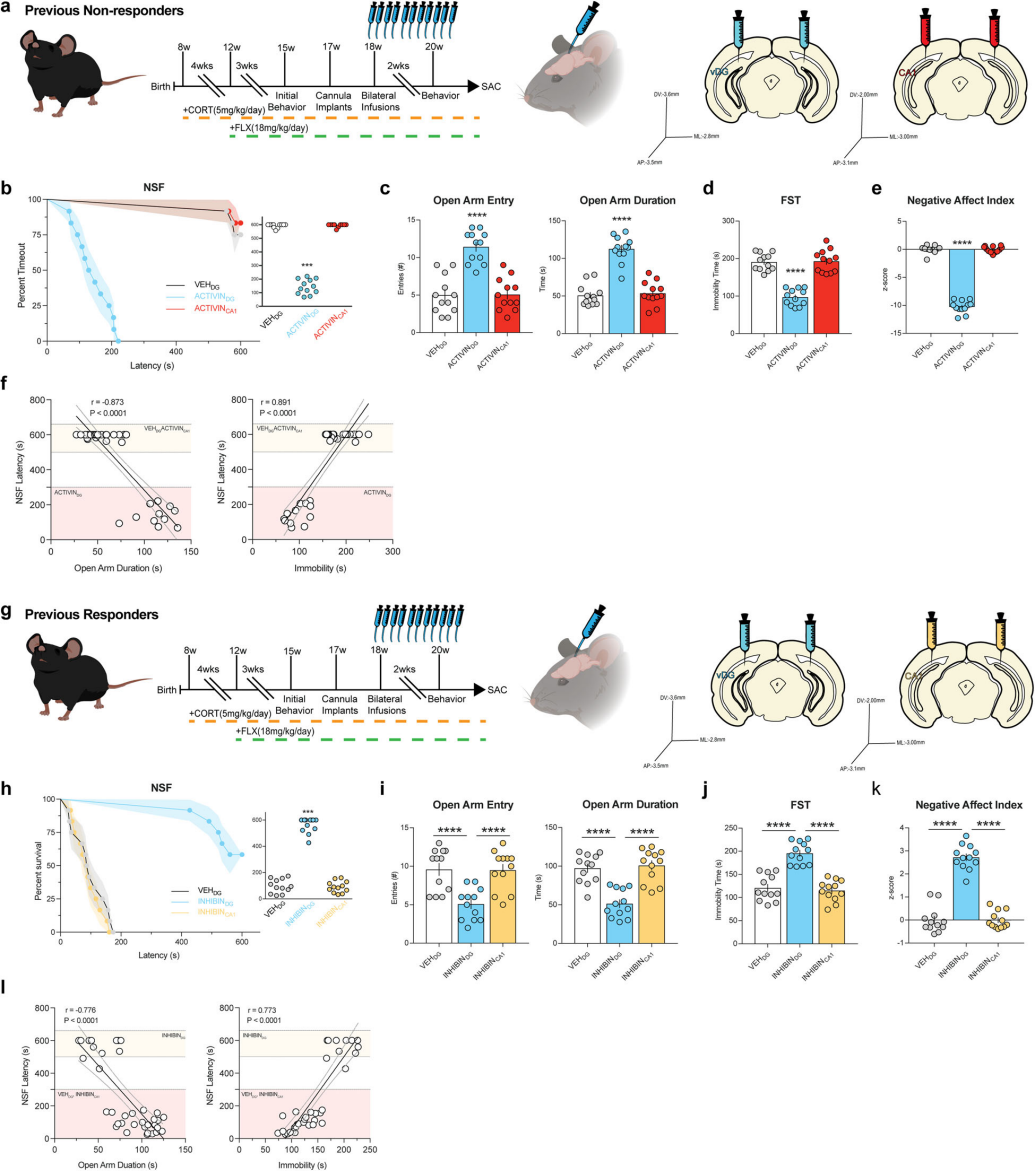
Chronic Activin A infusions into DG convert FLX non-responders into responders while chronic Inhibin A infusions convert responders into non-responders. **a** Timeline of experiment and coordinates of infusions for ventral DG and ventral CA1 infusions into FLX non-responders. **b** Kaplan–Meier survival curve (left panel) and scatterplot (right panel) of NSF data showing individual latency to eat values across all three FLX non-responder treatment groups: vehicle infusions into DG (VEH_DG_), Activin A infusions into DG (ACTIVIN_DG_), and Activin A infusions into CA1 (ACTIVIN_CA1_). **c**–**e** One-Way ANOVA of VEH_DG_, ACTIVIN_DG_, and ACTIVIN_CA1_ for: EPM open arm entries and open arm duration (**c**), FST immobility (**d**), and Negative Affect Index (**e**). **f** Regression analyses correlating NSF latency to eat with EPM open arm duration (left) and FST immobility (right). **g** Timeline of experiment and coordinates of infusions for ventral DG and ventral CA1 infusions into FLX responders. **h** Kaplan–Meier survival curve (left panel) and scatterplot (right panel) of NSF data showing individual latency to eat values across all three FLX non-responder treatment groups: vehicle infusions into DG (VEH_DG_), Inhibin A infusions into DG (INHIBIN_DG_), and Inhibin A infusions into CA1 (INHIBIN_CA1_). One-Way ANOVA of VEH_DG_, INHIBIN_DG_, and INHIBIN_CA1_ for: EPM open arm entries and open arm duration (**i**), FST immobility (**j**), and Negative Affect Index (**k**). **l** Regression analyses correlating NSF latency to eat with EPM open arm duration (left) and FST immobility (right). For survival curves, line shading shows SEM of each group (*n* = 12 per group). Scatterplots, horizontal lines, and bars show group means with error bars indicating SEM.

Next, since CORT+FLX response status persists for at least six months, we exposed a large cohort of C57BL/6J mice to CORT+FLX, and then non-responders (*n* = 36, see Supplemental Fig. 7 for initial NSF behavior) received bilateral cannula implants and were infused once daily for two weeks with either vehicle (0.1% BSA), Activin A peptide (1.0 μg per hemisphere) into DG, or Activin A peptide (1.0 μg per hemisphere) into CA1 (timeline in Fig. 4a). These mice were then exposed to NSF, EPM, and FST. Remarkably, CORT+FLX non-responders that received chronic Activin A infusions into DG had reduced latency to eat in the NSF relative to non-responders that received vehicle (*p* < 0.0001, log-rank Mantel–Cox test with Bonferroni correction) or chronic Activin A infusions into CA1 (*p* < 0.0001, log-rank Mantel–Cox test with Bonferroni correction) (Fig. 4b). Closer inspection of individual latencies demonstrated that all CORT+FLX non-responders that received DG Activin A infusions were converted into responders in the NSF. Group differences were also observed in the EPM for open arm entries (*F*_(2,33)_ = 31.6, *p* < 0.0001, Fig. 4c) and duration (*F*_(2,33)_ = 58.9, *p* < 0.0001, Fig. 4c) and in the FST for immobility (*F*_(2,33)_ = 57.4, *p* < 0.0001, Fig. 4d). CORT+FLX non-responders that received DG Activin A infusions had increased open arm entries and duration and decreased immobility relative to non-responders that received vehicle (*p* < 0.0001 for all, Bonferroni corrected) or chronic Activin A infusions into CA1 (*p* < 0.0001 for all, Bonferroni corrected). These data indicate that non-responders to CORT+FLX were converted into responders in NSF, EPM, and FST. The Negative Affect Index also demonstrated group differences (*F*_(2,33)_ = 647, *p* < 0.0001), with CORT+FLX non-responders that received DG Activin A infusions showing reduced negative affect relative to CORT+FLX non-responders that received vehicle or Activin A infusions into CA1 (*p* < 0.0001 for both, Bonferroni corrected) (Fig. 4e). Significant relationships also emerged when we directly compared NSF latency to feed to EPM open arm duration (Pearson *r* = −0.873, *p* < 0.0001), with linear regression line (*y* = −5.81*x*−864, *F*_(1,34)_ = 109, *p* < 0.0001), and to FST immobility (Pearson *r* = 0.891, *p* < 0.0001), with linear regression line (*y* = 3.77*x*−160, *F*_(1,34)_ = 131, *p* < 0.0001) (Fig. 4f). Taken together, these data demonstrate that supplementing Activin signaling in DG can convert FLX non-responders into responders across NSF, EPM, and FST.

### Inhibition of Activin signaling in DG converts FLX responders into non-responders

Since chronic Activin A infusions into DG can convert FLX non-responders into responders, we next wanted to test whether DG Activin signaling was necessary for the behavioral response to FLX. Specifically, we sought to determine whether inhibition of Activin signaling in DG converts FLX responders into non-responders. Inhibin is an endogenously occurring protein complex that has nearly opposite biological effects to Activin^26^. Inhibin binds directly to Activin receptor complexes, where Activin and Inhibin act as mutual antagonists to each other^26^. Therefore, a cohort of C57BL/6J CORT+FLX responders (*n* = 36, see Supplemental Fig. 7 for initial NSF behavior) received bilateral cannula implants and were infused once daily for two weeks with either vehicle (0.1% BSA), Inhibin A peptide (1.0 μg per hemisphere) into DG, or Inhibin A peptide (1.0 μg per hemisphere) into CA1 (timeline in Fig. 4g). These mice were then exposed to NSF, EPM, and FST. Excitingly, CORT+FLX responders that received chronic Inhibin A infusions into DG had increased latency to eat in the NSF relative to non-responders that received vehicle (*p* < 0.0001, log-rank Mantel–Cox test with Bonferroni correction) or chronic Inhibin A infusions into CA1 (*p* < 0.0001, log-rank Mantel*–*Cox test with Bonferroni correction) (Fig. 4h). Group differences were also observed in the EPM for open arm entries (*F*_(2,33)_ = 12.9, *p* < 0.0001, Fig. 4i) and duration (*F*_(2,33)_ = 24.1, *p* < 0.0001, Fig. 4i) and in the FST for immobility (*F*_(2,33)_ = 39.4, *p* < 0.0001, Fig. 4j). CORT+FLX responders that received DG Inhibin A infusions had decreased open arm entries and duration and increased immobility relative to responders that received vehicle (*p* = 0.0003 for open arm entries, *p* < 0.0001 for open arm duration and immobility, Bonferroni corrected) or chronic Inhibin A infusions into CA1 (*p* = 0.0003 for open arm entries, *p* < 0.0001 for open arm duration and immobility, Bonferroni corrected). The Negative Affect Index also demonstrated group differences (*F*_(2,33)_ = 120, *p* < 0.0001) (Fig. 4k), with CORT+FLX responders that received DG Inhibin A infusions showing increased negative affect relative to CORT+FLX responders that received vehicle or Inhibin A infusions into CA1 (*p* < 0.0001 for both, Bonferroni corrected). Significant relationships also emerged when we directly compared NSF latency to feed to EPM open arm duration (Pearson *r* = −0.776, *p* < 0.0001), with linear regression line (*y* = −5.99*x*−748, *F*_(1,34)_ = 51.3, *p* < 0.0001), and to FST immobility (Pearson *r* = 0.864, *p* < 0.0001), with linear regression line (*y* = 4.46*x*−391, *F*_(1,34)_ = 100, *p* < 0.0001) (Fig. 4l). Importantly, coinfusion of Inhibin A and Activin A also blocked the effects of Activin A on behavior (Supplemental Fig. 8). Taken together, these data demonstrate that Activin signaling in the DG is necessary for the behavioral effects of FLX treatment as directly inhibiting Activin signaling in DG converts FLX responders into non-responders across NSF, EPM, and FST. Furthermore, these data demonstrate that FLX behavioral response status can be bidirectionally modified by manipulating DG Activin signaling.

### Activin A infusions into DG are a more effective augmentation therapy than commonly used second-line treatments

**Fig. 5:**
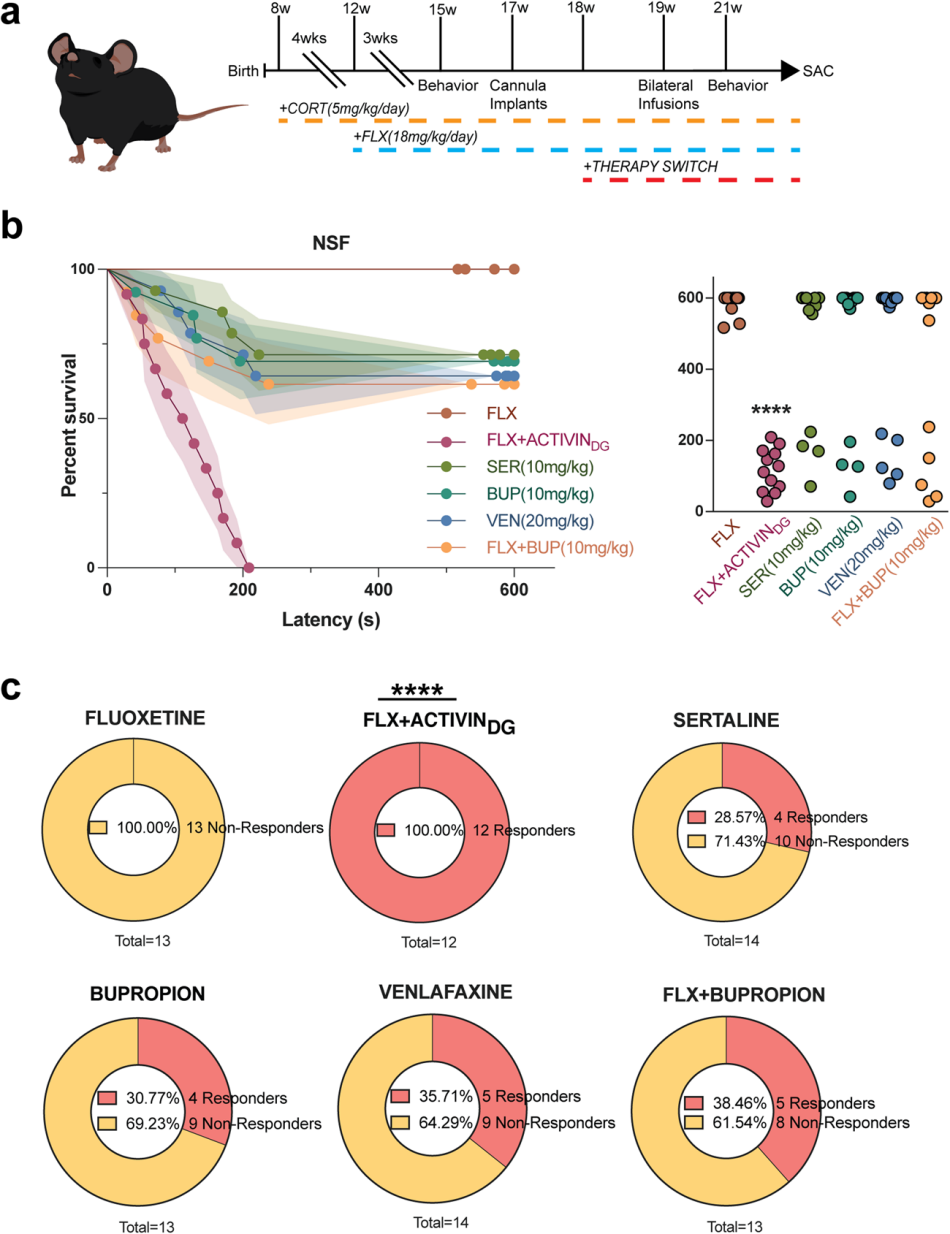
Activin A infusions into DG are a more effective augmentation therapy than commonly used second-line treatments. **a** Timeline of experiment. **b** Kaplan–Meier survival curve (left) and scatterplot (right) of NSF data showing individual latency to eat values across all six CORT+FLX non-responder treatment groups: CORT+FLX (FLX), CORT+FLX switched to CORT+FLX+Activin A into DG (FLX+ACTIVIN_DG_), CORT+FLX switched to CORT+sertraline (SER), CORT+FLX switched to CORT+bupropion (BUP), CORT+FLX switched to CORT+venlafaxine (VEN), and CORT+FLX switched to CORT+FLX+bupropion (CORT+FLX+BUP). **c** Graphical depiction of proportion of CORT+FLX non-responders converted into responders following different second-line antidepressant treatments.

When human patients do not remit to initial antidepressant therapy, they are usually switched to a new antidepressant. For example, in the large NIMH funded STAR*D study^2^, patients were first treated with citalopram (Celexa, a SSRI). Approximately 33% were found to display remission of depression symptoms. The 67% that did not remit were then subdivided into several groups and switched to either sertraline (Zoloft, a SSRI), bupropion (Wellbutrin, a norepinephrine/dopamine reuptake inhibitor), or venlafaxine (Effexor, a serotonin/norepinephrine reuptake inhibitor). Other groups either remained on citalopram and were augmented with bupropion or received other treatments. Our data suggest that chronic DG Activin A infusions are a very effective augmentation strategy for non-responders to FLX treatment. Therefore, we next wanted to assess whether switching mice from FLX to other antidepressants or augmenting FLX with other antidepressants is as effective in converting non-responders into responders as augmenting FLX with Activin A infusions into DG. To this end, we exposed a large cohort (*n* = 250) of C57BL/6J mice to chronic CORT+FLX and then assessed their behavior in NSF, where we found that *n* = 79 were non-responders to FLX treatment (see Supplemental Fig. 7 for initial NSF behavior). Two weeks later, we cannulated all 79 FLX non-responders to bilaterally target the DG and then housed the mice two per cage with a divider (timeline of experiment in Fig. 5a). One week after cannulation, we subdivided these non-responders into 6 groups of mice (*n* = 12–14 per group). Two groups remained on FLX, one group was switched to sertraline (SER, 10 mg/kg/day)^47^, one group was switched to bupropion (BUP, 10 mg/kg/day)^48^, one group was switched to venlafaxine (VEN, 20 mg/kg/day)^49^, and the remaining group remained on FLX but also began receiving bupropion (FLX+BUP, 10 mg/kg/day of BUP). Then, one week after the groups were formed, we began bilateral infusions. One of the two groups that remained on FLX alone received Activin A infusions, while the other five groups received vehicle infusions. Infusions were given once per day (over a time course of 15 min per hemisphere) for two weeks. We then retested these six groups of mice in NSF (Fig. 5b). The mice that remained on FLX only and received vehicle infusions remained non-responders. Consistent with the results in Fig. 4, 100% (12/12) of the mice that remained on FLX alone and received Activin A infusions into DG showed decreased latency to eat and were converted into responders (*p* < 0.0001 relative to FLX alone, log-rank Mantel–Cox test with Bonferroni correction) (Fig. 5b, c). By contrast, only 28.5% (4/14) of the mice switched to sertraline (*p* = 0.0408 relative to FLX alone, log-rank Mantel–Cox test, not significant with Bonferroni correction), 30.8% (4/13) of the mice switched to bupropion (*p* = 0.0329, log-rank Mantel–Cox test, not significant with Bonferroni correction), 35.6% (5/14) of the mice switched to venlafaxine (*p* = 0.0193, log-rank Mantel–Cox test, not significant with Bonferroni correction), and 38.5% (5/13) of the mice that remained on FLX and were augmented with bupropion (*p* = 0.0145, log-rank Mantel–Cox test, not significant with Bonferroni correction) were converted into responders. Therefore, only the chronic DG Activin A infused group showed a significantly decreased latency to eat in NSF relative to the FLX only group. These data strongly suggest that direct modulation of Activin signaling in the DG may be a more effective strategy for FLX non-responders than those that are currently used.

## Discussion

### Antidepressant treatment resistance

Within the United States, approximately 16% of the population will experience an episode of major depression in their lifetime^1^. Although commonly used treatments, such as SSRIs, are prescribed to relieve symptoms, only a subset of patients (~33%) achieve remission with initial treatment^3^. Given that SSRIs are also prescribed widely for several anxiety disorders and obsessive-compulsive disorder, this treatment resistance results in clinicians using decision-tree medical algorithms, such as the Texas Medication Algorithm Project (TMAP)^50^, in attempts to combat mood disorders in patients that do not remit to initial lines of treatment. As patients move through different levels of these treatment algorithms, remission rates drop dramatically (~35% with second treatment to ~16% with fourth treatment)^2,51,52^. Therefore, failure to achieve remission within two treatments results in very poor outcomes. These issues plague modern psychiatry and indicate that drugs targeting monoaminergic systems have reached a limit in terms of effectiveness. Much research now focuses on developing drugs that take aim at distinct targets, including glutamate modulators, anticholinergic agents, and opioid modulators rather than monoaminergic systems^53–58^. However, it remains unclear why SSRIs and other monoaminergic drugs are only effective for a subset of patients. Our unique approach here to assess SSRI treatment resistance in mice suggests that individual molecular differences within the neural circuitry underlying the antidepressant response may underlie response status. Direct activation of Activin signaling in the DG proved to be more effective in converting non-responders to FLX treatment into responders. Therefore, similar directed molecular or even neural circuit-based approaches may ultimately prove to be more effective augmentation strategies than blindly switching treatments.

### DG is a critical component of the neural circuitry mediating the antidepressant response

We assessed molecular differences between FLX responders and non-responders in the DG of mice exposed to chronic stress. Our data suggest that manipulation of DG Activin signaling can bidirectionally alter the behavioral response to FLX. These data further support a growing preclinical literature utilizing ablation, genetic, and neural circuit-based approaches to demonstrate that the DG is a principal component of the neural circuitry regulating both mood and the antidepressant treatment response^8,11,13,14,16–20^.

Several distinct populations of cells in the DG, including the young adult born granule cells (younger than 8 weeks)^9,11,13^, mature granule cells (developmentally born or adult born granule cells older than 8 weeks)^8^, and cytocholecystokinin (CCK)-positive GABAergic interneurons^20^ are implicated in mediating the antidepressant treatment response. In all likelihood, these distinct populations work in concert via local microcircuitry. One common thread among these cell types is that the young adult born granule cells and CCK-positive interneurons provide an inhibitory influence over the mature granule cells in the ventral DG that is critical for both stress resilience and the antidepressant response^17,19,20^. Furthermore, inhibitory 5-HT1A receptors on mature granule cells are required for the antidepressant response^8^. Consistently, Activin infusions increased adult neurogenesis and decreased DG neuronal activity as measured by cFos following NSF exposure (Supplemental Fig. 6). This decrease in DG activity likely results in decreased activation of ventral hippocampal outputs, including areas such as the medial prefrontal cortex and bed nucleus of the stria terminalis that form a neural circuit mediating affective behaviors^59^.

Our preliminary microarray data that implicated Activin signaling components in the antidepressant response and all data in this manuscript were from microdissections of the granule cell layer (GCL) of the ventral DG^41^, which is primarily composed of densely packed mature granule cells and sparse young adult born granule cells^60^. Therefore, we hypothesize that the Activin signaling components are being altered within these cell types. However, it will be important for future work to detail the exact effects of Activin signaling on DG neuronal ensembles, and how Activin signaling affects DG activity and ultimately other components of the neural circuitry underlying the antidepressant response.

### The role of Activin signaling in the antidepressant response

The importance of Activin and Inhibin signaling, as part of the TGF-β superfamily, is well-understood in the context of development, where Activin plays important roles in erythroid cell differentiation, induction of the dorsal mesoderm, and craniofacial development^26,61,62^. Activin also plays an essential role in pituitary follicle-stimulating hormone (FSH) production, while Inhibin inhibits FSH production^26,61^. However, the roles of these protein complexes are less understood in the context of the developed brain. While basal levels are low, Activin A is rapidly induced in the hippocampus by electroconvulsive seizures and long-term potentiation (LTP)-inducing high-frequency stimulation, where it plays a role in the maintenance of long-term memory and late-LTP^63–66^. Activin A and Acvr1A mRNA are upregulated in the DG by chronic treatment with the antidepressant paroxetine and Smad2 phosphorylation is induced by fluoxetine treatment^21,22^. Environmental enrichment (EE) induces Activin A mRNA expression in DG and CA3 and increases Smad2/3 phosphorylation in the hippocampus^66^. Overexpression of a dominant-negative Acvr1B in mouse forebrain under the CamKIIα promoter resulted in decreased avoidance in the Open Field and Light-Dark tests, a decreased behavioral response to benzodiazepines, enhanced spontaneous GABA release, and increased GABA tonus^67^. Inducible transgenic expression of Activin A under the CamKIIα promoter resulted in decreased avoidance in the Open Field, EPM, and Light-Dark Tests, while expression of Follistatin, an inhibitor of Activin signaling, under the CamKIIα promoter increased avoidance^27^. Furthermore, acute Activin A infusions into DG but not CA1 or Amygdala, reduces immobility in FST^21,22^. Acute Activin B infusions had no effect. Finally, a human genetic association study found 166 single nucleotide polymorphisms (SNPs) within 10 genes belonging to the Activin signaling pathway as being associated with antidepressant treatment response^22^. Genetic variants in the betaglycan gene (*TGFBR3*), a member of the human Activin system, showed the best association, as homozygote carriers of the major allele were significantly more frequent among the responders to antidepressant treatment^22^. Interestingly, we found differences in Smad2 and Smad3, where Smad3 was increased selectively in responders and Smad2 was selectively increased in non-responders. Therefore, future studies will need to assess the transcriptional targets of the Smad complex in responders and non-responders. Taken together with our results, it is now clear that Activin signaling in the DG is a critical component of the behavioral response to antidepressant treatment.

One important limitation of this study is that we only used male mice. Unfortunately, both chronic CORT administration and standard forms of chronic social defeat stress are not effective in female mice^46,68,69^. However, recent variations of social defeat may be useful for assessing the role of Activin signaling in the antidepressant response in female mice^69,70^. It is especially important for future studies to use females given that disruptions in peripheral Activin signaling can impact both male and female sex organs causing changes in secretion of sex hormones in both sexes^71^.

Our results demonstrate that FLX response status is conserved across several affective behavior tests (mice that show a response to FLX in NSF also show a response in EPM and FST and vice versa for non-responders) regardless of whether chronic corticosterone or chronic social defeat stress was used to induce a maladaptive affective state. Furthermore, FLX response status was highly correlated with the DG expression of multiple Activin signaling components. Functionally, chronic activation of Activin signaling in the DG successfully converted behavioral non-responders to FLX into responders. By contrast, chronic inhibition of Activin signaling in the DG converted responders to FLX into non-responders. This bidirectional modification is the first evidence that response or resistance to an antidepressant can be altered. Furthermore, these results strongly suggest that Activin signaling in the DG is a necessary component of achieving a behavioral response to antidepressant. Finally, chronic activation of Activin signaling proved to be a more effective second-line strategy for non-responders to FLX than several commonly used treatments.

## Supplementary information

Supplemental Text

Supplemental Figure 1

Supplemental Figure 2

Supplemental Figure 3

Supplemental Figure 4

Supplemental Figure 5

Supplemental Figure 6

Supplemental Figure 7

Supplemental Figure 8
