# Supplementary material for: Dentate gyrus activin signaling mediates the antidepressant response
Source: Transl Psychiatry. 2021 Jan 7;11:7. doi: 10.1038/s41398-020-01156-y (PMC7791138; doi:10.1038/s41398-020-01156-y)
Supplement: Supplementary file 1 — Supplemental Text [file 41398_2020_1156_MOESM1_ESM.docx]

SUPPLEMENTARY TEXT

SUPPLEMENTARY MATERIALS AND METHODS

***Drug administration***. Animals were either placed on chronic doses of either vehicle, which consisted of 0.45% β-cyclodextrin (Sigma-Aldrich) in water, or corticosterone (5mg/kg) (Sigma-Aldrich) dissolved in vehicle for duration of experiments. After 4 weeks of either vehicle or corticosterone administration, a subgroup of animals received either vehicle (autoclaved water) or fluoxetine hydrochloride (18mg/kg) (Biotrend) via daily oral gavage for throughout the experiment. On the days when mice were subjected to behavioral testing, fluoxetine or vehicle administrations were conducted after the mice completed the testing in order to avoid any acute effects. For additional therapies mice were then treated for 3 weeks with either: bupropion hydrochloride (10mg/kg) (Sigma-Aldrich), venlafaxine hydrochloride (20mg/kg) (Sigma-Aldrich), sertraline hydrochloride (10mg/kg) (Sigma-Aldrich), or fluoxetine hydrochloride (18mg/kg) (Biotrend) + bupropion hydrochloride (10mg/kg) (Sigma-Aldrich) via oral gavage.

***Behavioral testing***. Behavioral testing was conducted after 3 weeks of antidepressant administration, in the following order: EPM, NSF and then FST. Mice were given 3 days between behavioral tests to avoid contaminating stressors as well as before sacrifice. Prior to each behavioral test mice were acclimated to the room for 30 mins of habituation.

*Elevated plus maze*. EPM was performed as previously described^1^. The plus maze consisted of two closed arms and two open arms 2 feet above the floor. Mice were placed into the central area facing one closed arm and allowed to explore the maze for 5 mins. Data were scored using Ethovision software (Noldus), in which open arm time and open arm entries were recorded. Between animals, the maze was cleaned with 70% ethanol between each run.

*Novelty-suppressed feeding*. NSF was performed as described^1, 2^. The testing apparatus consisted of a plastic box (50 × 50 × 20 cm), the floor of which was covered with approximately 2 cm of bedding. Mice were weighed and food deprived 18 hrs before behavioral testing. At the time of testing, a single pellet of food was placed on a white paper platform in the center of the box beneath a gooseneck lamp illuminating the center of the area at about 1500 lux. A mouse was placed in a corner of the box and latency to approach and eat the food pellet was recorded with a maximum of 10 mins. Immediately after the testing period, the mice were transferred to their home cages and latency to feed in the home cage as well as consumption was recorded. After completing testing, animals were weighed again and % change in body weight was calculated with respective to pre-testing weights.

*Forced swim test*. As previously described^1^, mice were placed into clear plastic buckets 20 cm in diameter and 23 cm deep, filled two-thirds of the way up with 26 °C water and were videotaped. Mice were in the forced swim buckets for 6 mins, but only the last 4 mins were scored. Scoring was automated using Videotrack software (ViewPoint).

***Social Defeat Stress***. Paradigm was conducted as previously described. In brief, defeat stress was carried out using similar methods to those already published^3, 4^. Prior to social defeat stress, male retired breeder CD1 mice (Charles River Labs) were screened for aggression. CD1 mice that attacked screener mice (C57/BL6J) for two consecutive days in less than 60 seconds were selected. After selection of aggressors, experimental mice were exposed to a different CD1 aggressor mouse each day for 5 min over 10 days. After contact, experimental mice were inspected for injury and separated from the aggressor and placed in an adjacent compartment of the same cage as the CD1 mouse, separated by a plastic divider with holes. Control test mice were housed in equivalent cages but with members of the same strain, which were changed daily. Twenty-four hours after the last session, all mice were housed individually for the remainder of the study. Following 10 days of social defeat stress, animals were run through a social interaction test in which mice were placed in an open field for 2.5 minutes for baseline exploration in the absence of a novel CD1, and then for another 2.5 minutes in the presence of a CD1. Mice were deemed susceptible if they spent less time in the interaction zone when the social target was present than absent AND a total time spent interacting with the social target <30s, and resilient if the they spent more time in the interaction zone when the social target was present than absent AND total time spent interacting with the social target >60s.

***Gene Expression***. Animals were sacrificed via rapid decapitation and dentate gyrus was microdissected, flash frozen, and then stored at -80°C until further processing. RNA was extracted from tissue samples using a RNA/DNA Purification kit (Norgen Biotek). Total RNA was then converted into cDNA using Superscript III enzyme (Invitrogen). Quantitative-PCR was performed in triplicate reactions with Taqman Fast Advanced Mastermix and Taqman probes for activin a, acvr1a, acvr1b, acvr1c, smad2, smad3, and rn18s (Life Technologies) on a StepOne Plus Real-Time PCR System (Applied Biosystems). Data was analyzed using the ΔΔC_T_ method, triplicate cycle thresholds per gene per sample were averaged, normalized to control gene (*rn18s*) to obtain ΔC_T_, and were then converted to ΔΔC_T_ values by normalizing to mean ΔC_T_’s of the vehicle group. Final values were then expressed as an expression percentage relative to the vehicle group values.

***Intracerebral infusions****.* Mice were anesthetized with sodium pentobarbital (diluted 1:10 from stock of 50mg/ml and injected at a volume of 10 ml/kg) (Henry Schein) and guide cannulae with dummy cannulae (Plastics 1) were implanted. For ventral DG the coordinates used were: -3.5 mm and ±2.8 mm from bregma at a depth of 3.6mm from the skull surface, and for CA1 the coordinates used were: -3.1mm and ±3.0mm from the bregma at a depth of 2.0mm from the skull surface. 1-2 weeks after surgery, animals began to receive bilateral infusions of either vehicle (0.1% BSA) (Sigma-Aldrich), 1μg of mouse Activin A peptide (R&D Systems), 1μg of mouse Inhibin A peptide (R&D Systems), or 1μg each of Activin A + Inhibin A (R&D Systems) in vehicle once per day (over a time course of 15 minutes per side, 10 minutes of infusion and an additional 5 minutes with tubing left in place) for 2 weeks prior to behavioral testing. Each day connector assemblies with tubing were connected to internal cannulae, which were then inserted into the guide cannulae. Infusions were delivered by a standard infusion only syringe pump (Harvard Apparatus). A total volume of 1.0 μl was infused in each hemisphere per day. Animals were freely moving in their cage during infusions.

SUPPLEMENTARY FIGURE LEGENDS

**Supplemental Figure 1** *NSF data for DG mRNA expression cohort of mice and serum FLX levels* (a) Timeline of experiment. (b) Kaplan-Meier survival curve (large panel) and scatterplot (small panel) of NSF data showing individual latency to eat values across all four treatment groups. (c) Two-way ANOVA of all treatment groups (small panel) and One-Way ANOVA of CORT+VEH, CORT+FLX responders and CORT+FLX non-responders (large panel) for serum FLX levels following three weeks of FLX administration. For survival curves, line shading shows SEM of each group (n=15-23 per group). Scatterplots, horizontal lines, and bars show group means with errors bars indicating SEM.

**Supplemental Figure 2** No differences in homecage consumption for all experiments. (a-c) One-way ANOVAs showed no significant differences in homecage consumption across groups used to assess behavioral differences in treatment response to FLX. (d-e) Experiments involving infusion of activin (d) or inhibin (e) did not impact homecage consumption following NSF behaviors. (f) Infusions of activin, inhibin, or co-infusions did not impact homecage consumption. (g) Application of different antidepressants did not impact homecage consumption following exposure to NSF.

**Supplemental Figure 3** *Identification and classification of susceptible mice in Figures 3 and 4* (a) Timeline of experiment and diagram of CSDS and Social Interaction (SIT) protocol. (b) Time spent in social interaction zone reveal significant difference (*F*_(3,67)_ = 106, p < 0.0001) only in the presence of a CD1 aggressor. The only difference between groups was CNTRL animals compared to SUS+VEH (p < 0.0001, Bonferroni-corrected), SUS+FLX-R (p < 0.0001, Bonferroni-corrected), or SUS+FLX-NR (p < 0.0001, Bonferroni-corrected). (b) Social interaction ratios were significantly different (*F*_(4,66)_ = 62.2, p < 0.0001). The only difference between groups was CNTRL animals compared to SUS+VEH (p < 0.0001, Bonferroni-corrected), SUS+FLX-R (p < 0.0001, Bonferroni-corrected), and SUS+FLX-NR (p < 0.0001, Bonferroni-corrected).

**Supplemental Figure 4** *Dose Response Used to Select Dose for Intracerebral Infusions.* (a) One-way ANOVA for VEH, 0.25ug, 0.5ug, 1ug, and 5ug showed no effects in dosages for open field distance traveled. (b-c) One-way ANOVA showed no impact of different dosgaes on EPM behavior in open arm entry or duration. (d) Kaplan-Meier survival curve (left) and scatterplot (right) of NSF data showing individual latency to eat values across all five treatment groups. Kaplan-Meier analyses revealed significant differences were observed between mice injected with VEH and those injected with 5ug (*x*(1)=6.74, p=0.0094), with 5ug having a lower latency to eat. (e) No differences were seen in home cage consumption following NSF behavior. (f) A One-way ANOVA, with Bonferroni corrected post hoc comparison, showed significant differences in FST immobility time (*F*_(4,55)_ = 6.202, p=0.0003). Mice injected with 1ug or 5ug spent less time immobile than VEH (1ug: p=0.019; 5ug: p=0.0132) and 0.25ug (1ug: p=0.0105; 5ug: p=0.0072) injected mice.

**Supplemental Figure 5** *Behavioral Differences Between BSA or Activin Treated Mice.* (a) 2x2 ANOVA revealed no effect of CORT or treatment (BSA or ACTIVIN) on distance traveled in the open field. (b & c) Separate two-way ANOVAs showed a significant interaction between CORT and treatment group in EPM open arm entries (b, *F*_(1,39)_ = 5.219, p=0.0279) and duration (c, *F*_(1,39)_ =6.38, p=0.0157). Separate Bonferroni corrected posthoc comparisons showed BSA+CORT mice entered less and spent less time on the open arms than BSA+VEH (entries: p=0.0256; duration: p=0.0018), ACTIVIN+CORT (entries: p=0.0853; duration: p=0.0073), and ACTIVIN+VEH (duration: p=0.0034) mice. (d) Kaplan-Meier survival curve (left) and scatterplot (right) of NSF data showing individual latency to eat values across all four treatment groups. Kaplan-Meier analysis revealed significant differences in the four groups latency to eat (*x*(3)=21, p=0.0001), with Bonferroni corrected Kaplan-Meier tests showing that ACTIVIN infusion significantly decreases latency to eat compared to BSA treatment (BSA+VEH vs ACTIVIN+VEH *x*(1)=6.36, p=0.0116; BSA+CORT vs ACTIVIN+CORT *x*(1)=12.17, p=0.0005). (d) Two-way ANOVA showed no differences in homecage consumption across the groups. (e) 2x2 ANOVAs revealed a significant effect of treatment (BSA or ACTIVIN) on FST immobility time (*F*_(1,39)_ = 13.56, p=0.0007), with Bonferroni post-hoc comparisons revealing VEH+BSA mice spent more time immobile than ACTIVIN+VEH (p=0.046) and ACTIVIN+CORT (p=0.038) mice.

**Supplemental Figure 6** *Effects of Activin infusions on DG* (a) Nissl staining of ventral DG from a CORT + Activin infused mouse. Location of cannula is indicated. (b) Two-way ANOVA revealed a significant effect of treatment (BSA or ACTIVIN) on DG BrdU+ cells (*F*_(1,39)_ = 20.59, p<0.0001), with Bonferroni post-hoc comparisons revealing ACTIVIN+CORT had higher numbers of BrdU+ cells than BSA+VEH (p=0.0012) and BSA+CORT (p=0.0005) mice. (c) A two-way ANOVA (left) showed significant effects of treatment (*F*_(1,28)_=30.67, p<0.0001) and CORT (*F*_(1,28)_ = 4.57, p=0.0413) on DG DCX+ cells with Bonferroni post-hoc comparisons revealing ACTIVIN+CORT had more DCX+ cells than BSA+VEH (p<0.0001) and BSA+CORT (p<0.0001) mice. An example of a DCX DAB-stained DG section showing DCX+ cells and dendrites from a CORT + Activin mouse is also provided (right). (d) A two-way ANOVA illustrated that a significant interaction between treatment and CORT (*F*_(1,20)_ = 4.69, p=0.042) on DG cFos expression, with Bonferroni post-hoc comparisons revealing ACTIVIN+CORT had less DG cFos expression than BSA+VEH (p=0.0191) and BSA+CORT (p=0.0001) mice. Additionally, ACTIVIN+VEH mice had less DG cFos expression than BSA+CORT (p=0.0008) mice.

**Supplemental Figure 7** *Initial NSF data showing initial behavior of non-responders used for infusion experiments* (a-c) and application of different antidepressant classes (d). There were no differences in initial behavior across groups.

**Supplemental Figure 8** *Coinfusion of Activin A and Inhibin A into Non-Responders blocks the effects of Activin A on behavior* (a) Timeline of experiment and coordinates of infusions for ventral DG. (b) Kaplan-Meier survival curve (large panel) and scatterplot (small panel) of NSF data showing individual latency to eat values across all four non-responder treatment groups: VEH, Activin A infusions into DG (ACTIVINDG), Inhibin A infusions into DG (INHIBINDG), and combined Activin A and Inhibin A infusions into DG (ACTIVIN+INHIBINDG), with all ACTIVINDG vs VEH, ACTIVINDG vs INHIBINDG, ACTIVINDG vs ACTIVIN+INHIBINDG p < 0.0001 for all, logrank Mantel-Cox test with Bonferroni correction. (c-e) One-Way ANOVA of VEH, ACTIVINDG, INHIBINDG, and ACTIVIN+INHIBINDG for EPM open arm entries (c left panel, F(3,44) = 27.3, p < 0.0001), EPM open arm duration (c right panel, F(3,44) = 34, p < 0.0001), FST immobility (d, F(3,44) = 48.2, p < 0.0001), and Negative Affect Index (e). **** indicates p < 0.0001 for all one-way ANOVA Bonferroni posthocs. (f) Regression analyses correlating NSF latency to eat with EPM open arm duration (left) and FST immobility (right). For survival curves, line shading shows SEM of each group (n=12 per group). Scatterplots, horizontal lines, and bars show group means with errors bars indicating SEM.

SUPPLEMENTAL REFERENCES

1. Samuels BA, Anacker C, Hu A, Levinstein MR, Pickenhagen A, Tsetsenis T *et al.* 5-HT1A receptors on mature dentate gyrus granule cells are critical for the antidepressant response. *Nature Neuroscience* 2015; **18**(11)**:** 1606-1616.

2. Samuels BA, Hen R. Novelty-Suppressed Feeding in the Mouse. In: Gould TD (ed). *Mood and Anxiety Related Phenotypes in Mice: Characterization Using Behavioral Tests, Volume II*. Humana Press: Totowa, NJ, USA, 2011, pp 107-121.

3. Anacker C, Luna VM, Stevens GS, Millette A, Shores R, Jimenez JC *et al.* Hippocampal neurogenesis confers stress resilience by inhibiting the ventral dentate gyrus. *Nature* 2018; **559**(7712)**:** 98-102.

4. Tsankova NM, Berton O, Renthal W, Kumar A, Neve RL, Nestler EJ. Sustained hippocampal chromatin regulation in a mouse model of depression and antidepressant action. *Nat Neurosci* 2006; **9**(4)**:** 519-525.
