# Supplementary figures and images for: Dentate gyrus activin signaling mediates the antidepressant response
Source: Transl Psychiatry. 2021 Jan 7;11:7. doi: 10.1038/s41398-020-01156-y (PMC7791138; doi:10.1038/s41398-020-01156-y)

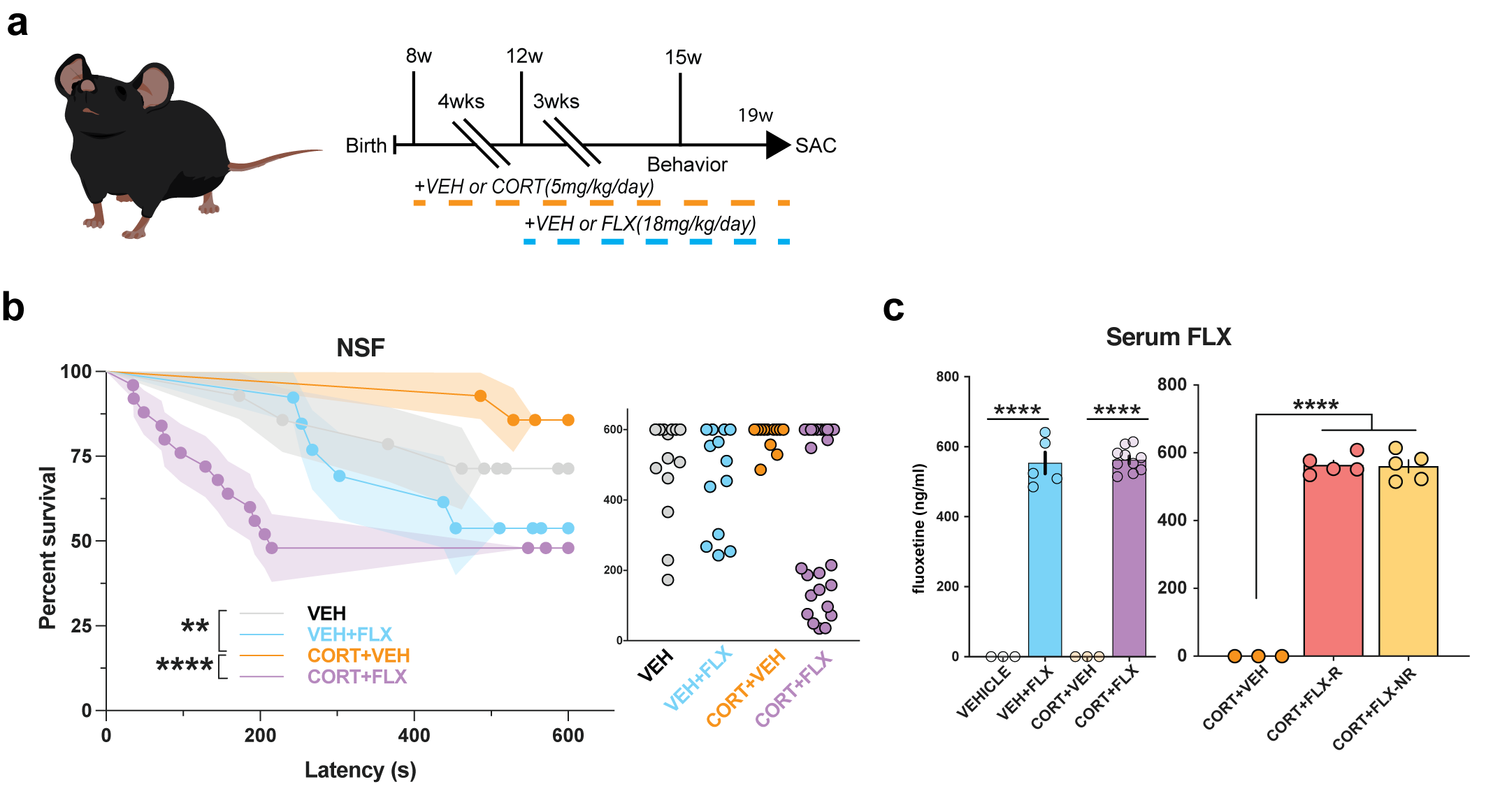

Supplement: Supplementary file 2 — Supplemental Figure 1 [file 41398_2020_1156_MOESM2_ESM.tif]

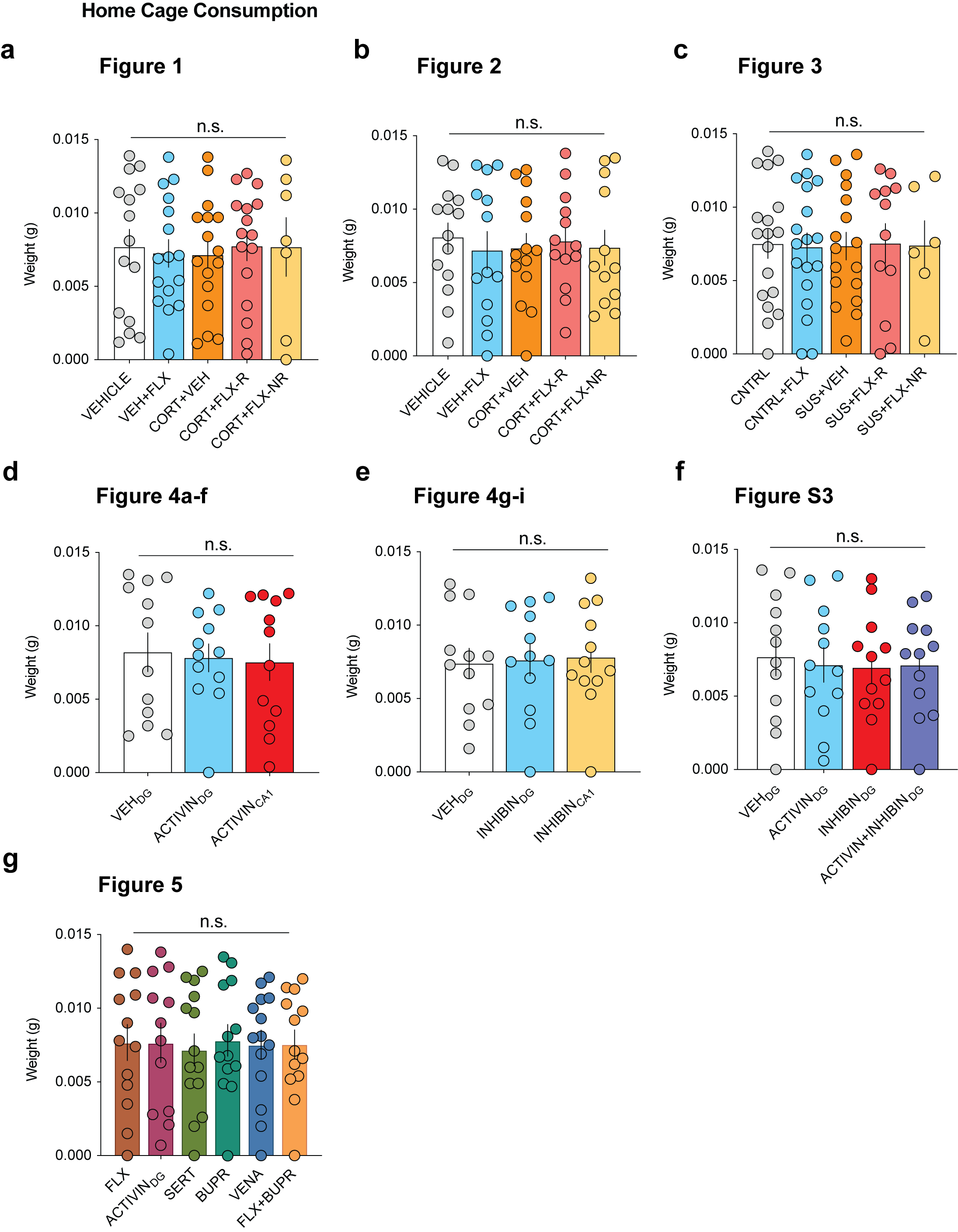

Supplement: Supplementary file 3 — Supplemental Figure 2 [file 41398_2020_1156_MOESM3_ESM.tif]

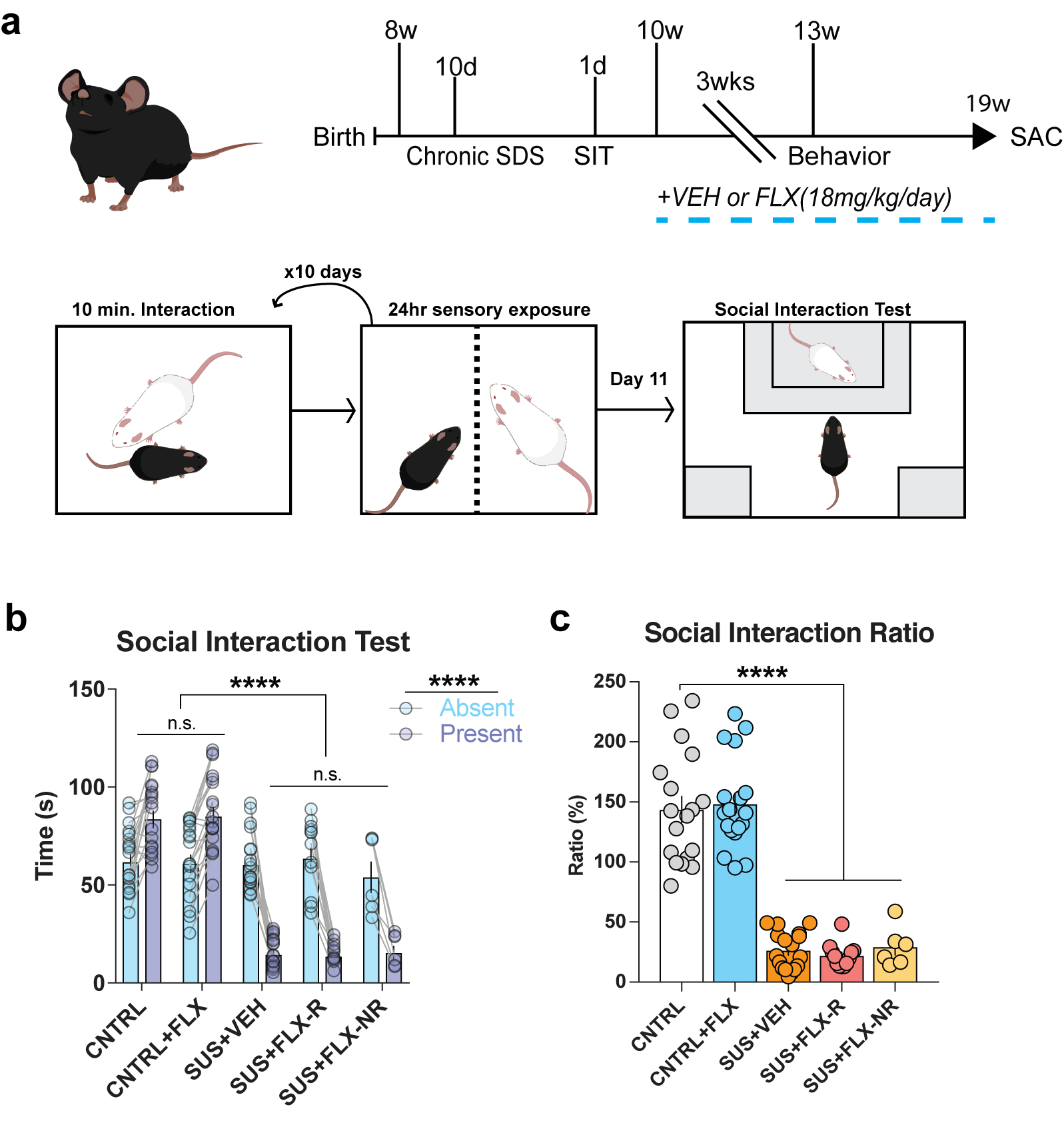

Supplement: Supplementary file 4 — Supplemental Figure 3 [file 41398_2020_1156_MOESM4_ESM.tif]

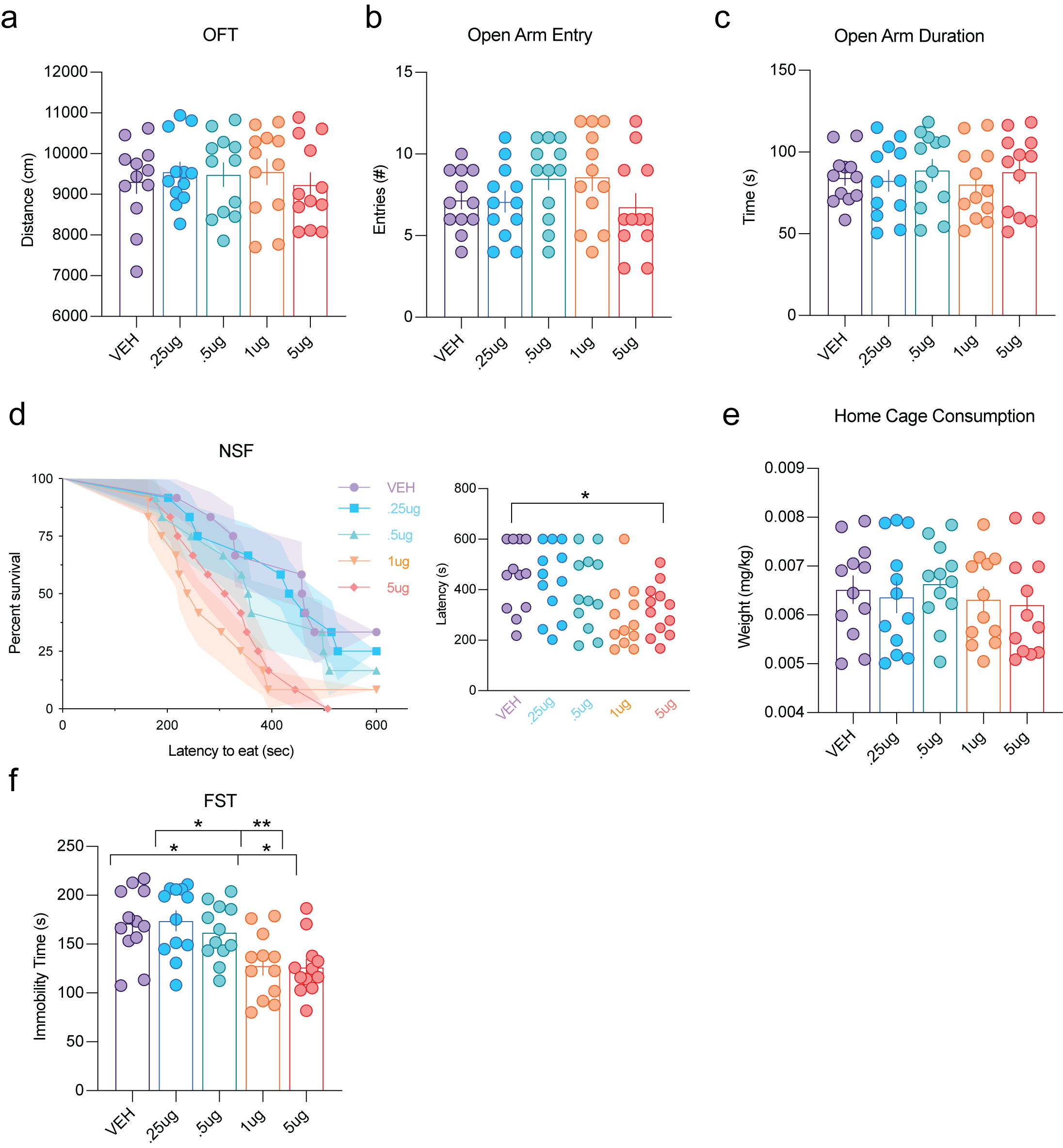

Supplement: Supplementary file 5 — Supplemental Figure 4 [file 41398_2020_1156_MOESM5_ESM.tif]

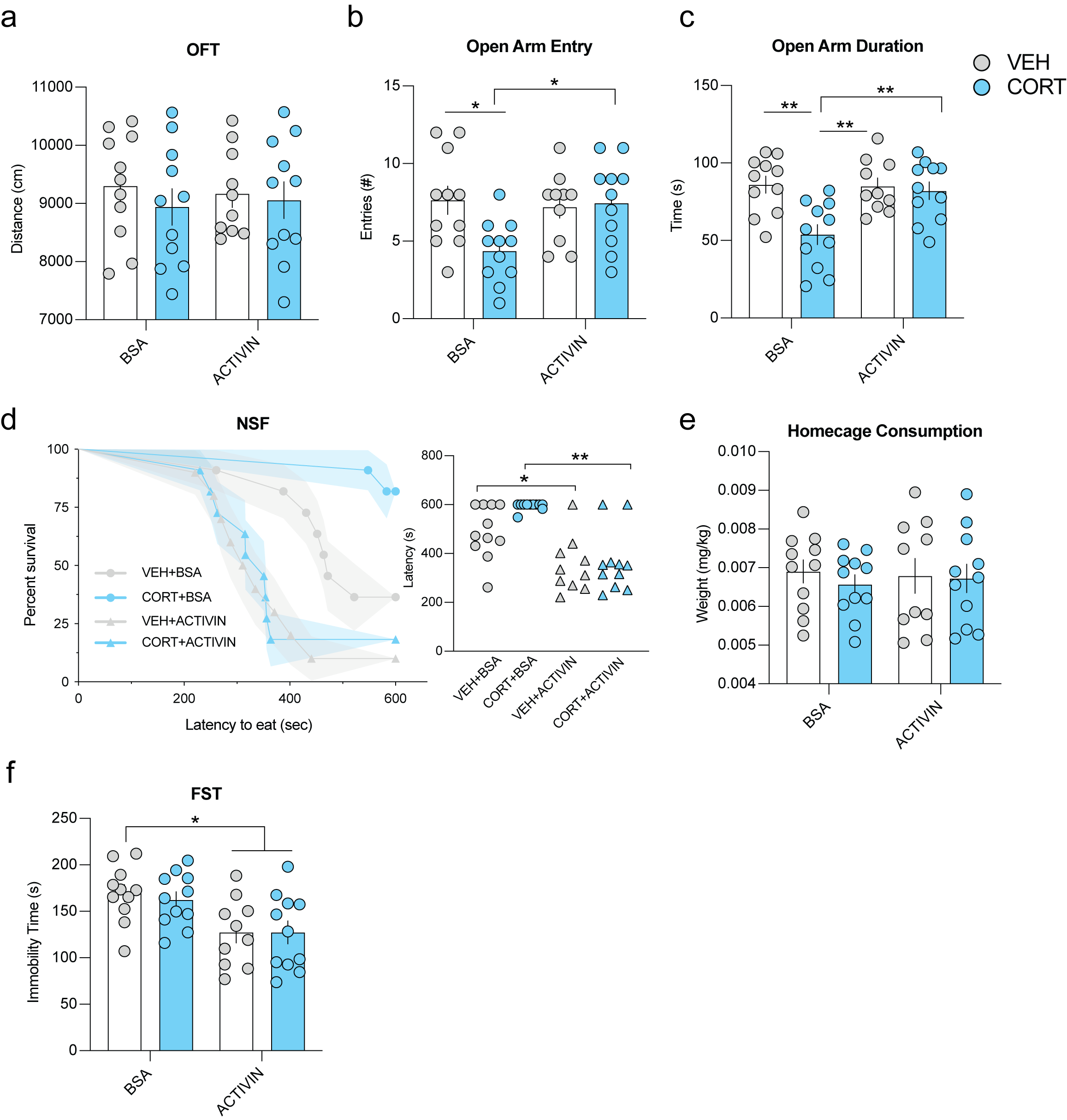

Supplement: Supplementary file 6 — Supplemental Figure 5 [file 41398_2020_1156_MOESM6_ESM.tif]

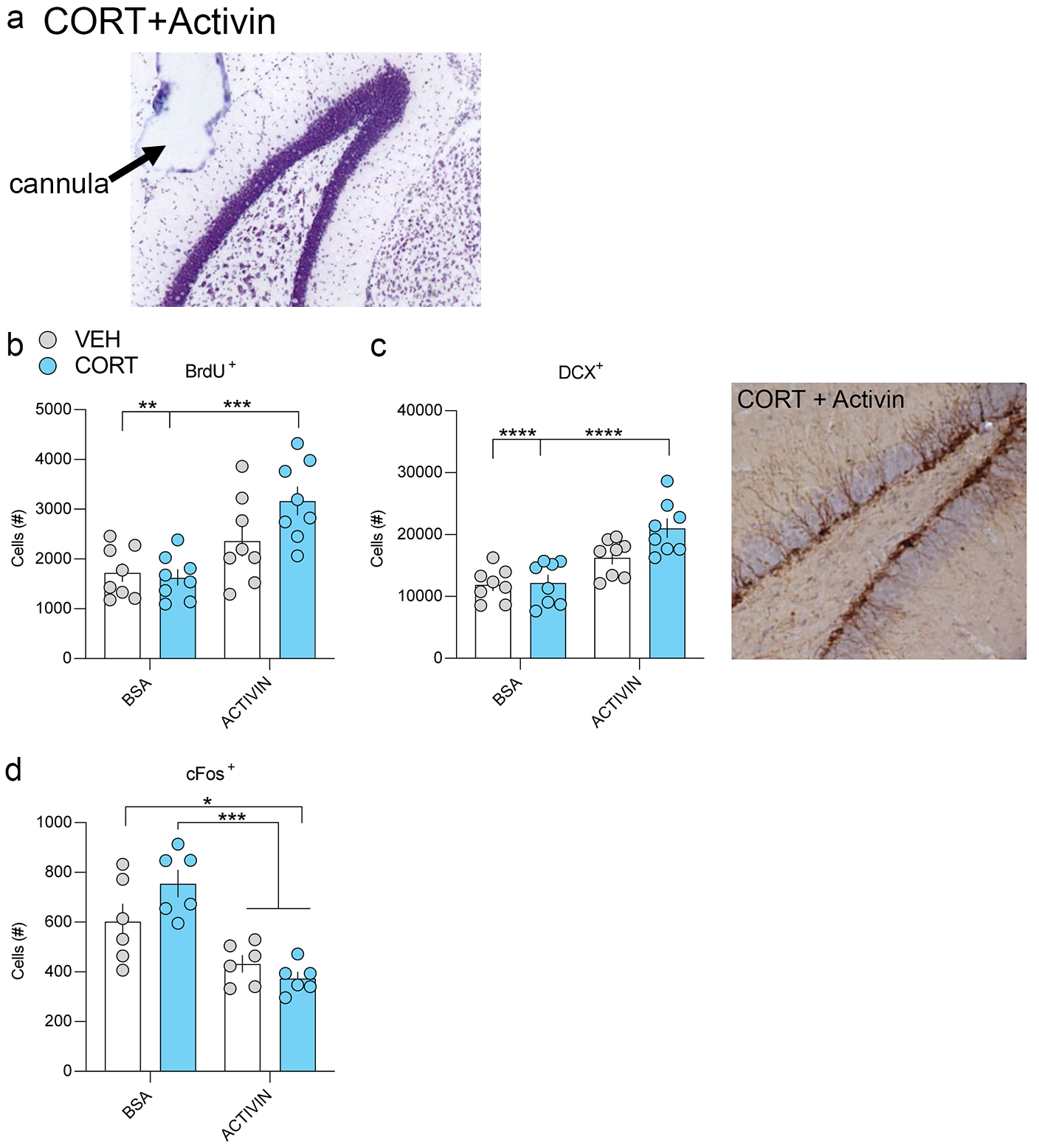

Supplement: Supplementary file 7 — Supplemental Figure 6 [file 41398_2020_1156_MOESM7_ESM.tif]

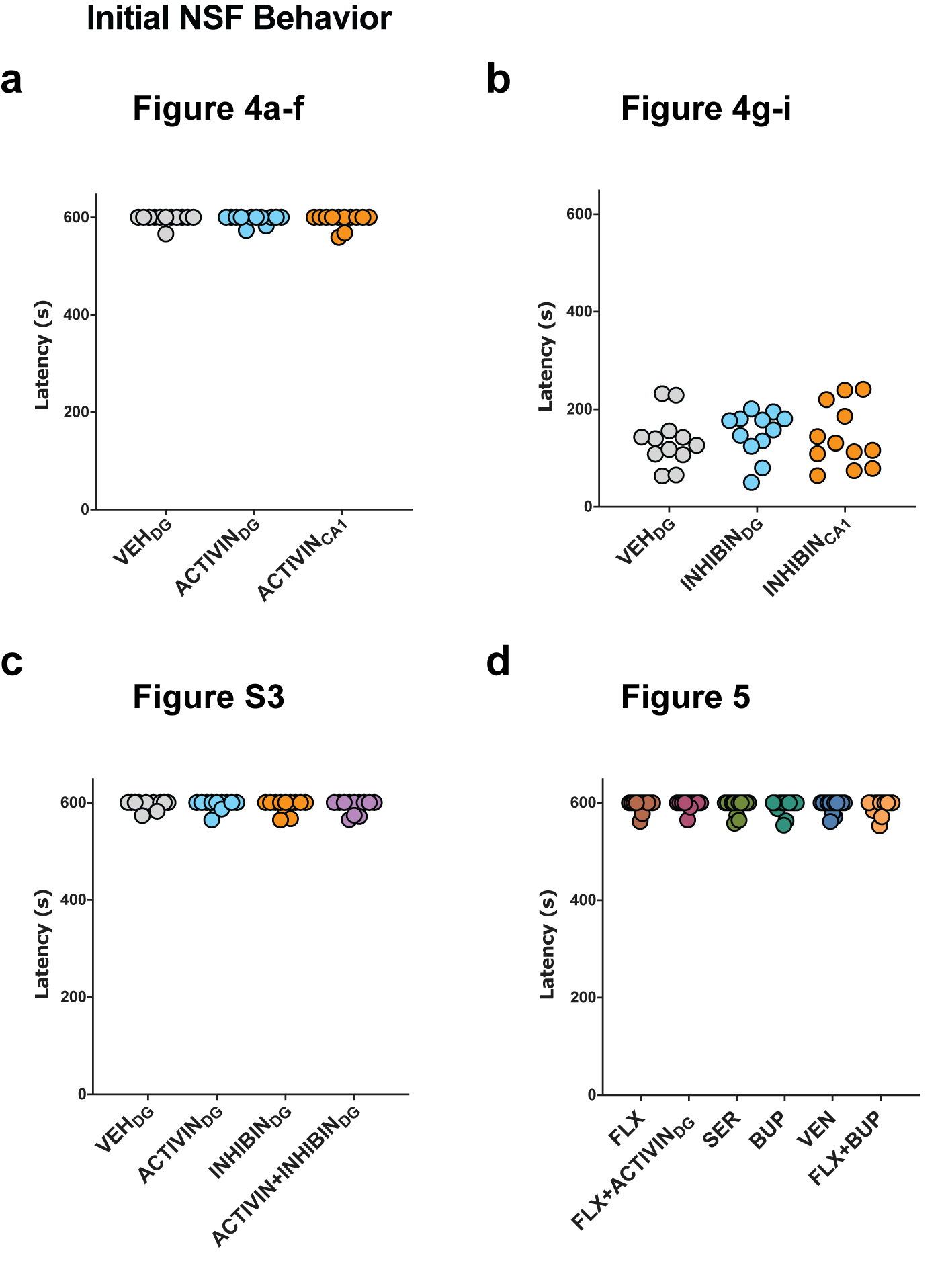

Supplement: Supplementary file 8 — Supplemental Figure 7 [file 41398_2020_1156_MOESM8_ESM.tif]

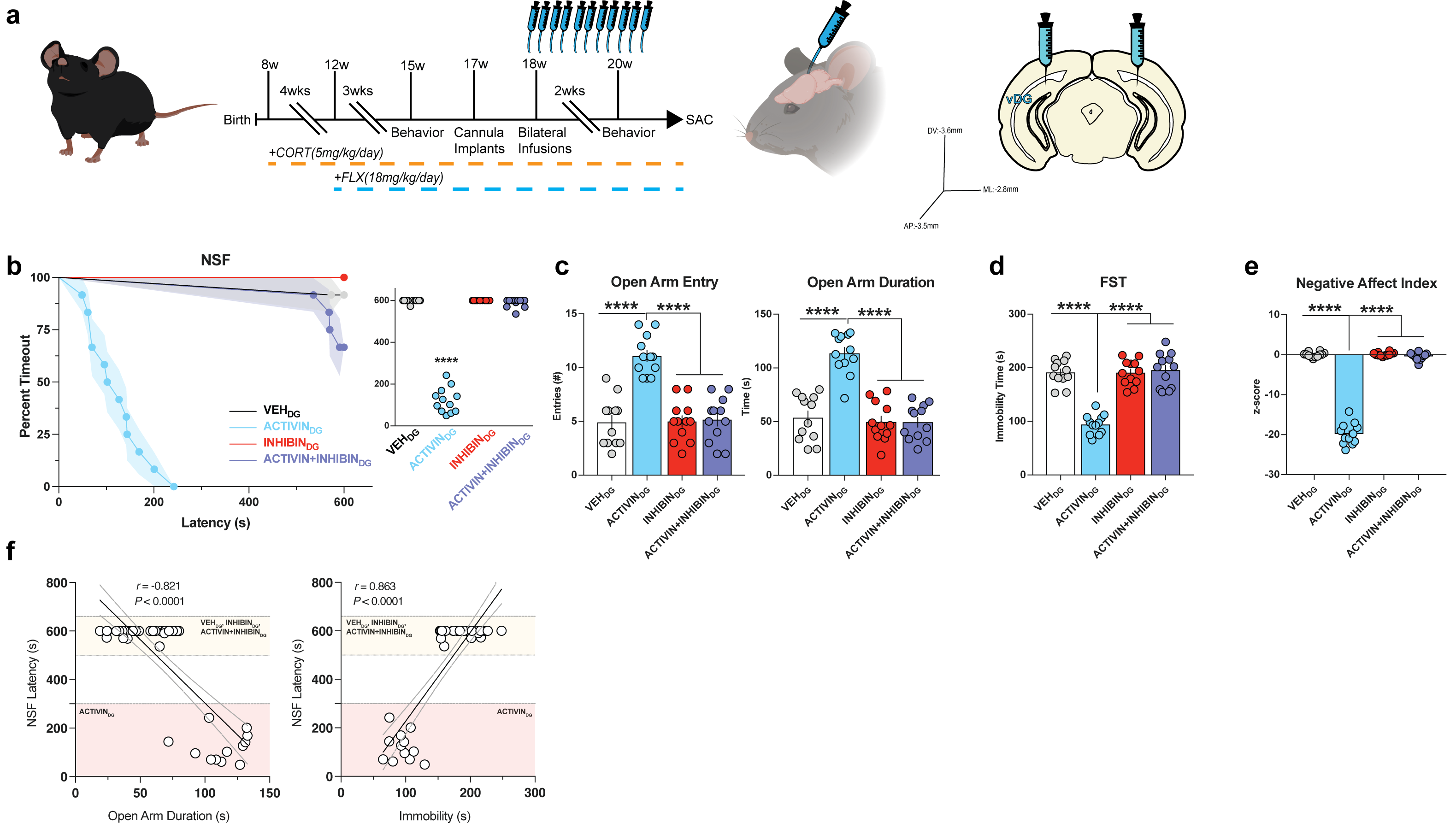

Supplement: Supplementary file 9 — Supplemental Figure 8 [file 41398_2020_1156_MOESM9_ESM.tif]
